# A functional genomics predictive network model identifies regulators of inflammatory bowel disease

**DOI:** 10.1038/ng.3947

**Published:** 2017-09-11

**Authors:** Lauren A Peters, Jacqueline Perrigoue, Arthur Mortha, Alina Iuga, Won-min Song, Eric M Neiman, Sean R Llewellyn, Antonio Di Narzo, Brian A Kidd, Shannon E Telesco, Yongzhong Zhao, Aleksandar Stojmirovic, Jocelyn Sendecki, Khader Shameer, Riccardo Miotto, Bojan Losic, Hardik Shah, Eunjee Lee, Minghui Wang, Jeremiah J Faith, Andrew Kasarskis, Carrie Brodmerkel, Mark Curran, Anuk Das, Joshua R Friedman, Yoshinori Fukui, Mary Beth Humphrey, Brian M Iritani, Nicholas Sibinga, Teresa K Tarrant, Carmen Argmann, Ke Hao, Panos Roussos, Jun Zhu, Bin Zhang, Radu Dobrin, Lloyd F Mayer, Eric E Schadt

**Affiliations:** 1 Department of Genetics and Genomic Sciences, Icahn School of Medicine at Mount Sinai, New York, New York, USA; 2 Icahn Institute of Genomics and Multi-scale Biology, Icahn School of Medicine at Mount Sinai, New York, New York, USA; 3 Sema4, a Mount Sinai venture, Stamford, Connecticut, USA; 4 Janssen Research and Development, LLC., Spring House, Pennsylvania, USA; 5 Department of Immunology, University of Toronto, Toronto, Ontario, Canada; 6 Department of Oncological Sciences, Tisch Cancer Institute, Icahn School of Medicine at Mount Sinai, New York, New York, USA; 7 Department of Pathology and Cell Biology, Columbia University Medical Center, New York, New York, USA; 8 Graduate School of Biomedical Sciences, Icahn School of Medicine at Mount Sinai, New York, New York, USA; 9 The Precision Immunology Institute, Icahn School of Medicine at Mount Sinai, New York, New York, USA; 10 Institute for Next-Generation Healthcare, Icahn School of Medicine at Mount Sinai, New York, New York, USA; 11 Division of Immunogenetics, Department of Immunobiology and Neuroscience, Medical Institute of Bioregulation, Kyushu University, Fukuoka, Japan; 12 University of Oklahoma Health Sciences Center, Oklahoma City, Oklahoma, USA; 13 Department of Comparative Medicine, University of Washington, Seattle, Washington, USA; 14 Department of Medicine (Cardiovascular Division), Albert Einstein College of Medicine, Bronx, New York, USA; 15 Thurston Arthritis Research Center and Department of Medicine, Division of Rheumatology, Allergy, and Immunology, University of North Carolina at Chapel Hill, Chapel Hill, North Carolina, USA; 16 Lineberger Comprehensive Cancer Center, University of North Carolina at Chapel Hill, Chapel Hill, North Carolina, USA; 17 Bristol-Myers Squibb, Research & Development, Pennington, New Jersey, USA; 18 Division of Clinical Immunology, Icahn School of Medicine at Mount Sinai, New York, New York, USA

## Abstract

A major challenge in inflammatory bowel disease (IBD) is the integration of diverse IBD data sets to construct predictive models of IBD. We present a predictive model of the immune component of IBD that informs causal relationships among loci previously linked to IBD through genome-wide association studies (GWAS) using functional and regulatory annotations that relate to the cells, tissues, and pathophysiology of IBD. Our model consists of individual networks constructed using molecular data generated from intestinal samples isolated from three populations of patients with IBD at different stages of disease. We performed key driver analysis to identify genes predicted to modulate network regulatory states associated with IBD, prioritizing and prospectively validating 12 of the top key drivers experimentally. This validated key driver set not only introduces new regulators of processes central to IBD but also provides the integrated circuits of genetic, molecular, and clinical traits that can be directly queried to interrogate and refine the regulatory framework defining IBD.

Crohn's disease and ulcerative colitis are the predominant forms of IBD and are characterized by relapsing and remitting inflammation of the intestine. While Crohn's disease and ulcerative colitis are marked by distinct clinical phenotypes and some overlap in molecular pathways, they largely have a shared genetic architecture. Despite GWAS having identified more than 200 IBD-associated loci thus far, these known genetic variants only contribute approximately 26% of Crohn's disease and 19% of ulcerative colitis heritability^1–6^. In susceptible individuals, the interaction of genetics with a wide range of environmental factors triggers a cascade of excessive and chronic inflammation, tissue damage, and impaired intestinal function.

For IBD, the construction of causal network models provides a way to organize large-scale, diverse data by statistically inferring causal relationships among any set of traits of interest, providing a comprehensive characterization of the architecture of disease. Genes associated with IBD susceptibility loci have been demonstrated to at least partially organize into coherent networks defining complex biological processes. In particular, IBD-related genes have been shown to organize into regulatory networks that are significantly enriched for immune and inflammatory processes. One such example was an immune-enriched network identified as enriched for genes associated with IBD susceptibility and immune function, and was suggestive of dynamic interactions relevant to IBD pathogenesis^2^. This network has been loosely associated with many different diseases, including obesity and diabetes, asthma, chronic obstructive pulmonary disease (COPD), and Alzheimer's disease^7–10^. However, thus far, no IBD network models have been proposed that are derived from the relevant molecular states of IBD, in IBD-relevant tissues, across different disease stages, which collectively reflect the full spectrum of disease.

Here we sought to integrate large-scale DNA and RNA variation data in the context of active IBD to construct a model of the pathological inflammatory component of IBD, which can aid in distinguishing between the inflammatory component causally associated with IBD and the homeostatic background function of the intestine. Using the existing state of knowledge around the immune network as a seed to construct instances of this model, we generated three causal networks defining IBD and identified the conserved inflammatory component (CIC) in each, which we consider as homologous networks given that they are highly conserved and derived from a single immune network seed.

These homologous intestine-derived CIC IBD networks were enriched for genes associated with known Crohn's disease and ulcerative colitis susceptibility loci contained within cell-type-specific epigenetic regulatory regions. Key driver genes (KDGs) predicted to modulate the regulatory states of these networks were identified and prioritized for experimental validation in a human macrophage cell system and mouse models of IBD to demonstrate the impact on IBD pathophysiology and for molecular validation of the network predictions. Our results not only validate the notion that perturbations in master regulators of the CIC IBD network model impact the pathogenesis of disease, but also elucidate how this model is regulated. The construction and validation of a predictive model that hierarchically organizes genomic and functional genomic data in an accessible way, and that identifies the components that modulate molecular states causally associated with IBD, is a first step in creating a more sustainable and accessible framework that leverages extensive data sets, enabling a more complete understanding of the regulatory components of IBD.

## Results

### Defining an immune gene seed set for the CIC IBD model

**Figure 1:**
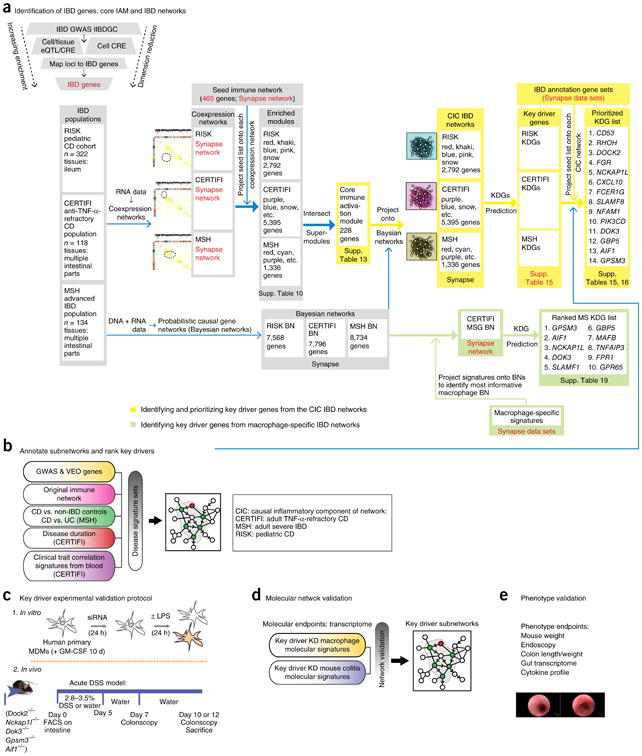
An integrative approach for constructing a predictive network model of IBD, and identifying and validating master regulators of these networks. (**a**) Identification of causal IBD genes. We identified IBD-associated DNA variants in immune cells and digestive-tissue-derived CRE regions, some of which also corresponded to eQTLs derived from patients with IBD. Identification of a core IAM. Three different populations representing distinct states of disease were profiled, and the resulting data were integrated to build predictive molecular networks of the intestine. The core IAM was derived from coexpression modules from pediatric and adult patients with moderate and advanced IBD who were screened to link the immune network to active IBD in a disease-relevant context. The core IAM from all three populations was identified as the most highly enriched for genes in the immune network but then also highly enriched for causal IBD GWAS genes and macrophage expression. (**b**) Identification and annotation of IBD networks. Subnetworks for each IBD cohort representing the core IAM were identified as different instances of the CIC IBD network model and annotated using a diverse set of data. Identification and ranking of KDGs. KDGs were identified from the CIC IBD networks and prioritized by the size of their effect on the network as well as the degree of disease subnetwork support. (**c**) KDG experimental validation. To validate the utility of the CIC IBD model as a regulatory framework for modeling IBD, the top KDGs were experimentally validated in IBD mouse models and human macrophage cell systems. (**d**) Molecular validation of the CIC IBD model. Signatures from mouse intestine and human macrophages were projected onto the CIC IBD model to evaluate the degree to which the model predicted KDG perturbation signatures. (**e**) Phenotype validations. *In vitro* and *in vivo* phenotypes of inflammation were measured to evaluate the functional role of the KDGs in the context of IBD. CD, Crohn's disease; UC, ulcerative colitis; KD, knockdown.

To construct the different representations of the CIC IBD model (Fig. 1), we identified a previously described macrophage-enriched immune network (referred to here as the immune network) that is not only enriched for IBD susceptibility genes and IBD-associated inflammatory processes^2^, but also has been implicated in a broad range of human diseases^9^. This immune network reflects the existing states of knowledge related to the immune component of the IBD network and so was chosen to serve as a seed set of genes that could be used to identify a homologous set of genes in an IBD-tissue-specific context. To define this IBD-tissue-specific context, we used gene expression data generated from intestinal tissues isolated from three independent populations of patients with IBD representing different stages of disease: treatment-naive pediatric patients (RISK cohort)^11^, patients refractory to anti–tumor necrosis factor (TNF)-α treatment who have participated in an ustekinumab clinical trial (CERTIFI cohort)^12,13^, and patients with advanced disease (novel MSH population) (Supplementary Table 1). A polygenic risk score was calculated on patients with IBD in the three independent populations, with no significant difference in risk score distributions across the adult and pediatric IBD populations detected (Supplementary Fig. 1 and Supplementary Table 2).

### Identifying causal IBD genes to annotate the CIC IBD network model

Genes assigned to reported IBD-associated loci are speculative^14^, and much of the variation in these loci resides in noncoding regions. Thus, we identified candidate causal IBD genes by integrating IBD risk SNPs, expression quantitative trait loci (eQTLs), and *cis*-regulatory element (CRE) data (Fig. 1a). Because of the highly context-specific nature of eQTLs^15^, we curated an IBD-focused data set of eQTLs derived from the RISK, MSH (Supplementary Table 3), and CERTIFI populations. In total, we identified a combination of genes associated with ulcerative colitis and Crohn's disease GWAS CRE expression SNPs (eSNPs) (IBD-associated eSNPs located in CREs) and CRESNPs (IBD-associated SNPs in CREs) (Supplementary Table 4 and Supplementary Note). CREs specific to innate immune cell types were found to have the highest amount of shared genetic architecture between Crohn's disease and ulcerative colitis (Supplementary Table 5). When comparing the expression of Crohn's disease and ulcerative colitis GWAS genes in data from non-inflamed and inflamed tissues across different intestinal regions from the CERTIFI population, we found distinct cell-type-specific enhancer regions significantly enriched for variants associated with Crohn's disease and ulcerative colitis (Supplementary Fig. 2 and Supplementary Tables 5–7).

For all cell types considered, the gene sets associated with the ulcerative colitis and Crohn's disease CRESNPs were assembled and intersected with modules in the coexpression networks generated from the MSH, CERTIFI, and RISK data sets (Fig. 1b and Supplementary Tables 8 and 9). We identified modules in these networks that were significantly enriched for the immune network and formed super-modules by taking the union of the tagged modules within each population-specific network (Supplementary Tables 10–12). By intersecting the super-modules, we identified a core immune activation module (IAM) (Supplementary Table 13). This core IAM represented a set of immune genes conserved across all IBD populations and was among the most enriched for genes in the immune network, for known IBD causal genes, and for macrophage-specific genes (Supplementary Table 14). Therefore, we considered the core IAM as the most highly informed seed set of genes obtained from an IBD-specific context from which to construct the CIC IBD network model. To support the assumption that the core IAM is specific to immune-related disorders such as IBD, we examined whether genes identified from large-scale schizophrenia GWAS were enriched in this module, given that schizophrenia has not been as significantly associated with immune and inflammation processes, unlike diseases such as IBD, asthma, COPD, and Alzheimer's disease. No significant enrichment of schizophrenia-associated genes was detected in the core IAM (fold enrichment = 1.09, Fisher's exact test, *P* = 0.48)^16^.

### Constructing the CIC IBD network model

We used the core IAM to derive specific representations of the CIC IBD model from three Bayesian networks we constructed across the three stages of disease (collectively referred to as the IBD networks). We constructed three independent, but homologous, probabilistic causal gene networks from intestinal tissues isolated from patients with IBD in each population (Fig. 1c). To identify each CIC IBD network instance, we projected the core IAM onto the RISK, MSH, and CERTIFI Bayesian networks. Each network projection consisted of overlapping nodes (genes) from the intersection of this core IAM seed set with all nodes in the respective Bayesian networks, identifying all nodes in each network within a path length of two of the nodes in this overlap, and then identifying the largest connected graph from this set of nodes and all associated edges.

### Identification and prioritization of key drivers of the CIC IBD networks

**Figure 2:**
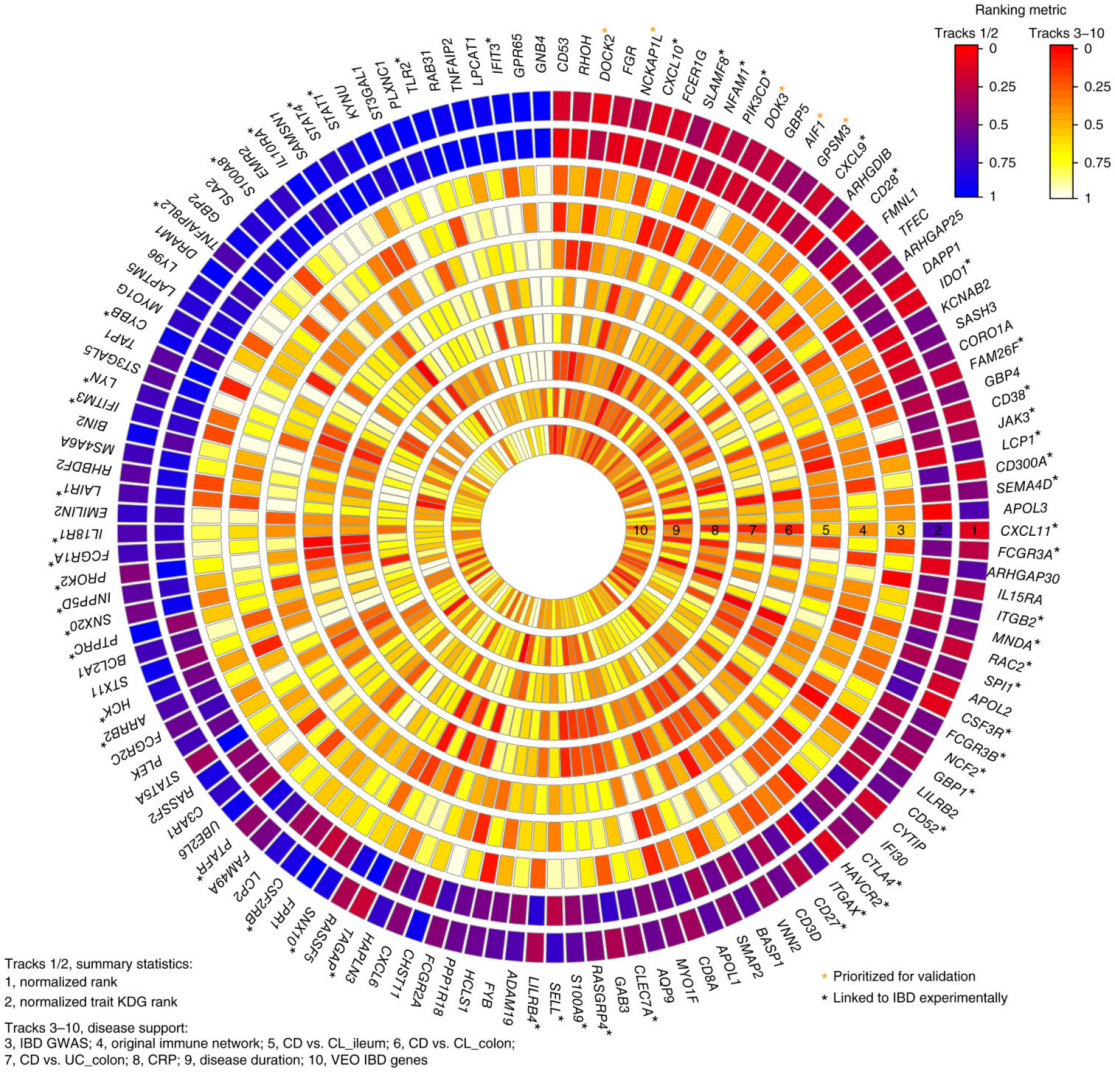
Ranking KDGs of the CIC IBD networks. One hundred and thirty-three KDGs were identified and scored with respect to genetic association to IBD, involvement in inflammatory processes associated with IBD, and association with IBD-related clinical traits. The KDGs are listed clockwise around the disk in rank order, starting with the top ranked KDG at the top of the disc. Each of the internal rings represents either a summary statistic used in the ranking or a type of disease support used to form the summary statistics for the ranking. The first two tracks are normalized rank and rank by disease trait enrichment, respectively, and the remaining tracks 3–10 are KDG rankings per trait (Supplementary Table 16): track 3, IBD GWAS; track 4, original immune network; track 5, Crohn's disease versus control (CL)_ileum; track 6, Crohn's disease versus CL_colon; track 7, Crohn's disease versus ulcerative colitis colon; track 8, CRP; track 9, disease duration; track 10, VEO IBD genes. The color depicted in each element signifies the degree of support provided by the trait rank for the corresponding KDG, with cooler colors reflecting weaker support and hotter colors reflecting stronger support. Black asterisks denote KDGs that have already been linked to IBD experimentally. Orange asterisks indicate prioritized KDGs for experimental validation.

To elucidate the regulatory framework of the CIC IBD model and its impact on IBD pathogenesis, we sought to identify and prospectively validate the master regulators predicted to modulate the state of the three CIC IBD networks. Using a previously defined KDG algorithm to determine these master regulators^10^, we identified 133 KDGs across all three intestinal CIC IBD networks (Supplementary Table 15). To prioritize these for experimental validation, we annotated them using four different categories of IBD-focused data sets: (i) genes identified in genetic studies as associated with IBD or very early onset (VEO) IBD; (ii) IBD-specific ileum and colon gene expression signatures from the MSH population; (iii) correlation signatures between clinical traits associated with IBD and gene expression data from the CERTIFI population; and (iv) the original immune network. We projected each of these IBD gene sets onto each of the CIC IBD networks and identified the KDG signatures most enriched for the IBD gene and trait signatures (Supplementary Tables 15 and 16). We then rank-ordered the 133 KDGs on the basis of a composite score that considered all lines of evidence supporting the KDGs in an IBD-informative context, thus providing a quantitative measure of the importance and degree of causal association each KDG had to IBD. From the top 10% of the KDGs in this rank-ordered list (Figs. 1d and 2, and Supplementary Table 15), we identified five KDGs that had not previously been validated as an IBD-associated gene: *DOCK2* (encoding dedicator of cytokinesis 2), *GPSM3* (G-protein-signaling modulator-3), *AIF1* (allograft inflammatory factor 1), *NCKAP1L* (NCK-associated protein 1 like), and *DOK3* (downstream of kinase 3).

Each of these five KDGs was predicted to be a master regulator of the network, given that each was predicted to significantly modulate the transcriptional state of the CIC IBD networks and thus impact the IBD-associated genes enriched in these networks, as well as effector-inflammation-associated traits. The KDGs we identified were all upregulated in the inflamed intestinal signatures but were not upregulated in the uninflamed intestinal signatures (Supplementary Table 7). In addition, they were all correlated with clinical variables such as disease duration, C-reactive protein (CRP), fecal calprotectin, and lactoferrin in blood and intestine (Supplementary Table 17).

According to published reports, the selected KDGs are primarily immune cell specific. *NCKAP1L*^17^, *DOCK2* (ref 18), and *AIF1* (ref 19) have roles in actin cytoskeleton organization via Rac activation, with *NCKAP1L* functioning in phagocytosis, migration, and formation of the immunological synapse for multiple immune cell types. *DOCK2*, an atypical guanine nucleotide exchange factor (GEF), has variants associated with immunodeficiency in T cells, B cells, and natural killer (NK) cells^20–25^. *DOCK2* and *AIF1* are also directly implicated in inflammatory cytokine secretion through RAC activation^26^. *GPSM3*, which has previously been linked to autoimmunity and chronic inflammation through GWAS^27^, is a negative regulator of the NLRP3 inflammasome affecting IL-1β levels^28^ and, like *AIF1*, impacts monocyte recruitment and cytokine secretion^19,29,30^. Finally, *DOK3*, a negative regulator of lipopolysaccharide (LPS) sensing through TLR4–ERK signaling in macrophages, regulates TLR3 signaling through TRAF3–TBK1–IRF3 and influences interferon (IFN)-β secretion^31–35^. Four of the KDGs we identified had not been experimentally linked to IBD but are disrupted in IBD pathology.

### Identification and prioritization of key drivers of the macrophage component of the IBD networks

Macrophages have a sentinel role in intestinal homeostasis and contribute to inappropriate inflammatory responses in IBD^36,37^. Previous studies^2^ have demonstrated that host–microbial interactions shape the genetic architecture of IBD. Given the macrophage enrichment^2^ of the original immune network and that the core IAM used to derive the homologous CIC IBD networks reflected this strong enrichment for macrophage-specific expression (Supplementary Tables 14 and 18), we more precisely identified this component of the IBD network.

The IBD networks were constructed from whole-tissue isolates involving a diversity of cell types but still largely reflected immune cell function and macrophage function in particular. However, the mixed cell types that constitute the IBD networks make it difficult to resolve the macrophage-specific components from the non-macrophage-specific components. To better resolve the macrophage component of these networks and identify KDGs serving as master regulators of this component, we leveraged a macrophage-specific gene signature (MSG; Supplementary Table 14). The CERTIFI CIC IBD network was the most enriched of the three CIC IBD networks for the macrophage signatures (Supplementary Table 18). To construct a more macrophage-specific IBD network, we projected genes in the MSG onto the CERTIFI IBD network and, from this projection, identified the largest connected subnetwork comprising nodes within a path length of three of these genes. We designated the resulting network as the macrophage-specific component of the IBD network.

To identify the master regulators of the macrophage component of the IBD network, we carried out KDG analysis and identified 133 KDGs, 59 of which were present in the independent human macrophage signature (MSS) gene set (Online Methods). Of these 59 genes, 38 were correlated with fecal calprotectin and differentially expressed in Crohn's disease and ulcerative colitis versus control disease signatures (Supplementary Table 19). Of these 38 KDGs, we identified 10 genes (including 4 of the 5 CIC IBD KDGs also included as macrophage KDGs) for experimental validation that were significantly correlated with clinical variables (Supplementary Table 17) and that have a role in macrophage function relevant to colitis: (i) *GPR65*, an IBD risk gene that has been shown to inhibit proinflammatory cytokine production in macrophages^38^; (ii) *GBP5*, which promotes NLRP3 inflammasome activation in response to pathogenic bacteria^39^; (iii) *MAFB*, a transcription factor controlling macrophage self-renewal^40^; (iv) *FPR1*, a gene involved in chemotaxis, phagocytosis, and reactive oxygen species (ROS) production in M1 macrophages^41^; (v) *SLAMF1*, a regulator of NADPH oxidase and phagolysosomal maturation^42^; and (vi) *TNFAIP3*, an IBD risk gene expressed in macrophage that inhibits the NLRP3 inflammasome^43^ and TNF-α-induced NF-κB. We identified an additional KDG, *LAPTM5*, a KDG in the macrophage component of the RISK network, given it is a macrophage-expressed gene known to modulate proinflammatory cytokine secretion in macrophages^44^.

To annotate the local network structure of the 11 macrophage KDGs, we identified the largest connected subnetwork in each CIC IBD network comprising these 11 KDGs, plus all genes within a path length of three of these KDGs. This macrophage-KDG-specific subnetwork was significantly enriched for IBD-associated macrophage/monocyte CRESNPs. Further, genes in this subnetwork downstream of the 11 KDGs are 2.72-fold (Fisher's exact test, *P* = 0.003) enriched for genes associated with monocyte and macrophage IBD CRESNPs (Supplementary Table 20). This statistically significant enrichment of macrophage IBD susceptibility genes was greater than the enrichment of macrophage-expressed genes in the MSS set, highlighting the predicted causal regulatory role of the macrophage KDGs in modulating genes linked to IBD susceptibility, beyond what was observed in genes that are expressed in macrophages. In addition, the increased enrichment of gene nodes in the macrophage KDG subnetwork for IBD macrophage and monocyte CRESNPs, as compared to IBD T cell CRESNP-associated genes, highlights the macrophage specificity of the CIC IBD networks.

### *In vitro* molecular network validation of the macrophage KDGs

**Figure 3:**
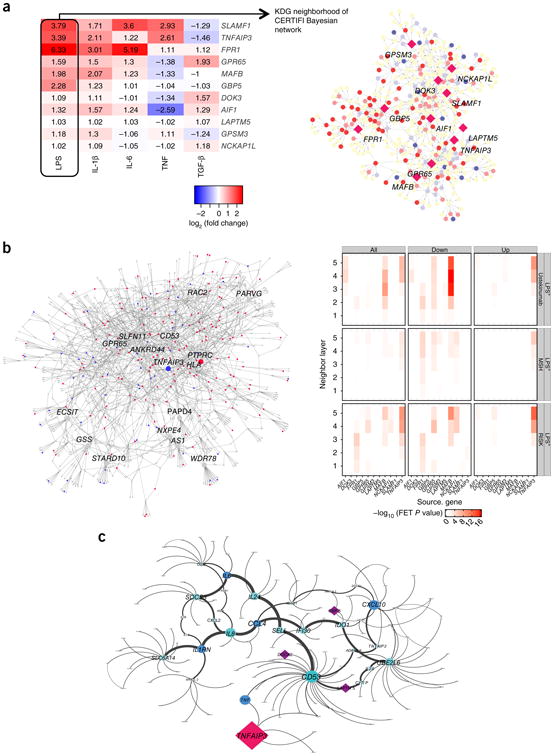
Transcriptional responses in stimulated macrophages to perturbations in macrophage KDGs are predicted by the IBD networks. (**a**) Table values represent fold change in expression of the indicated KDG in stimulated versus unstimulated macrophages. The network image illustrates the LPS-induced gene expression changes (red increased expression; blue decreased expression; intensity indicates magnitude of fold change) in the CERTIFI KDG (diamond nodes) network neighborhoods. (**b**) The heat map represents the −log_10_ *P* value for the enrichment of genes whose expression levels change in response to siRNA-mediated knockdown of the KDGs. The CERTIFI IBD network adjacent to the heat map is a representative example of genes that were upregulated (blue nodes) and downregulated (red nodes) in response to knockdown of the macrophage KDG *TNFAIP3*. The network-predicted TNFAIP3 signature was 1.84-fold enriched for genes in the *TNFAIP3* macrophage knockdown signature (Fisher's exact test (FET) *P* = 0.003). (**c**) Subnetwork of cytokines whose protein levels change in response to siRNA knockdown of *TNFAIP3* siRNA in macrophage. This subnetwork of differential protein cytokine expression contains *TNFAIP3* as well as other KDGs including *LAPTM5, DOCK2*, and *GBP5*. The red node represents the macrophage KDG *TNFAIP3*; blue nodes represent cytokines significantly differentially expressed in response to siRNA knockdown of *TNFAIP3*; purple nodes represent KDGs.

We performed molecular validation of the selected macrophage KDGs by profiling primary human monocyte-derived macrophages treated with non-targeting control small interfering RNA (siRNA) versus siRNAs targeting each of the 11 KDGs (Supplementary Fig. 3), under LPS stimulation conditions, given that LPS stimulation yielded the largest differential expression signature and the majority of the KDGs were responsive to LPS stimulation (Fig. 3a, Supplementary Table 21, and Supplementary Note). We found significant enrichment of the macrophage KDG-knockdown differential expression signatures in the networks. The macrophage-specific KDG-knockdown signature was well predicted by the corresponding KDG in the macrophage-specific component of the IBD network for 10 of the 11 macrophage KDGs tested. For example, the macrophage *MAFB*-knockdown signature was 2.2-fold enriched (Fisher's exact test, *P* = 6.1 × 10^−8^) in the macrophage-specific component of the network from which *MAFB* was originally identified. In addition, most of the knockdown signatures were significantly enriched within their respective KDG neighborhoods in the macrophage component of the other IBD networks as well as in the core IAM (Fig. 3b and Supplementary Tables 22 and 23).

### Experimental validation of KDG impact on inflammation

To assess the direct functional outcome of KDG perturbations in the macrophage, we measured cytokine levels in the supernatants of the LPS-stimulated macrophage with KDG knockdown versus non-targeted controls (Supplementary Fig. 4). We identified significant differential expression for IL-1RA and CXCL10 with *GPSM3* knockdown and for IL-6, CCL4, and CXCL10 with *NCKAP1L* and *FPR1* knockdown and found changes in IL-6, TNF-α, CCL4, and CXCL10 with *GPB5* knockdown, while *TNFAIP3* knockdown resulted in the strongest differential expression of cytokines (Fig. 3c). Many of these cytokines (or their receptors or ligands) have been associated with genetic susceptibility to IBD or have been suggested as drug targets for IBD, including TNF-α (ref 45), IL-6 (ref 46), IL-1RA^47^, CXCL10 (ref 48), and IL-12Rp40 (ref. 12) (Supplementary Table 24).

### *In vivo* molecular network validation of the intestinal KDGs

We sought to determine whether KDGs from the CIC IBD network model influence susceptibility to intestinal inflammation and to validate the KDGs *in vivo*. To this end, we employed a dextran sulfate sodium (DSS) mouse model of colitis. We performed RNA-seq profiling on distal colon tissue sampled from KDG-knockout and wild-type control animals exposed to DSS treatment. KDG gene expression signatures for each mouse model were constructed for DSS and baseline conditions by identifying genes that were differentially expressed between the knockout and wild-type animals. Each of the knockout differential expression signatures was enriched for molecular pathways relevant to IBD: the *Aif1*^−/−^ signature was 7.8-fold enriched for antigen receptor-mediated signaling genes (Fisher's exact test, *P* = 1 × 10^−7^); the *Gpsm3*^−/−^ signature was 6.2-fold (Fisher's exact test, *P* = 1 × 10^-5^) and 4.4-fold (Fisher's exact test, *P* = 0.00017) enriched for genes involved in the response to IFN-γ and TNF-α, respectively; the *Dock2*^−/−^ signature was 9.7-fold enriched for positive regulation for adenylate cyclase (Fisher's exact test, *P* = 0.00056), and the *Dok3*^−/−^ signature was 8-fold enriched for genes involved in the negative regulation of retinoic acid receptor (Fisher's exact test, *P* = 4.1 × 10^−12^). These knockout signatures were also enriched for IBD-related genes and clinical trait signature genes (Supplementary Tables 25 and 26). A single module (brown) was identified in a coexpression network generated from the mouse gene expression data (Supplementary Table 27) that was enriched for the KDG signatures, Crohn's disease GWAS and VEO IBD genes, and the core IAM, demonstrating a core conservation of the CIC IBD networks between species (Supplementary Fig. 5).

To determine whether the different IBD networks could accurately predict the genes that would change in response to perturbations of the KDGs, we tested whether the observed knockout signature for a given KDG significantly overlapped with the set of genes the network predicted would be under the control of that KDG (referred to as the KDG signature). The knockout signatures for *Gpsm3*, *Nckap1l*, *Dock2*, and *Aif1* were all significantly enriched in the corresponding KDG signatures predicted by the network, providing direct experimental validation of the network predictions (Supplementary Table 28). For example, *DOCK2* was identified as a KDG in the IBD networks and the differential expression signature in *Dock2*^−/−^ was 4.59-fold enriched (*P* = 7.91 × 10^−8^) for genes in the *DOCK2* signature predicted by the MSH IBD network. Overall, the experimental perturbation signatures for each KDG, with the exception of *Dok3* in the intestine, were significantly enriched for the network signature predicted by the IBD networks (Supplementary Fig. 6 and Supplementary Table 28).

**Figure 4:**
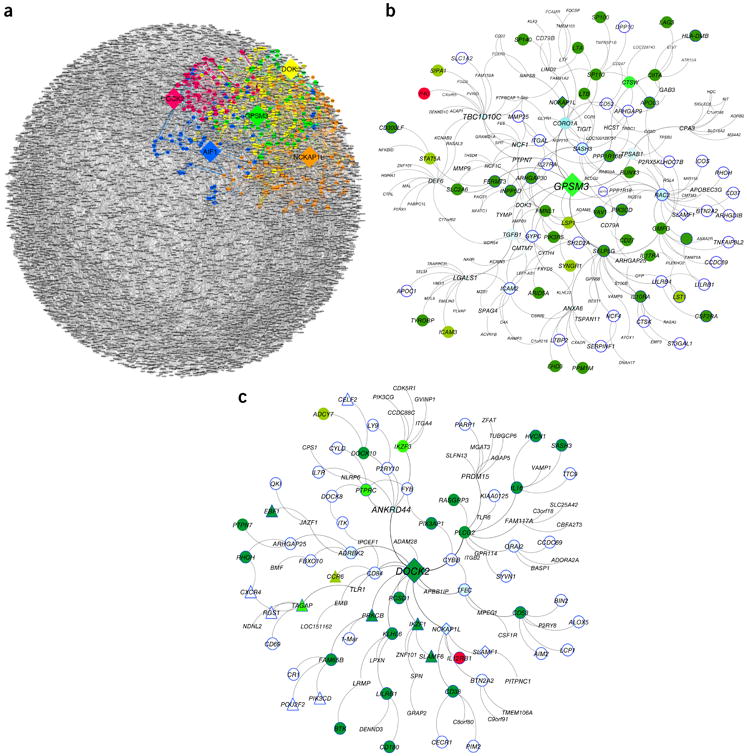
Enrichment analysis of KDG subnetworks. (**a**) Predicted transcriptional signature of 5 KDGs in the adult IBD networks. (**b**) The KDG (diamond) *GPSM3* subnetwork is 2.76-fold enriched (Fisher's exact test, *P* < 0.008) for monocyte and macrophage IBD CRESNPs (light green) and 3.58-fold enriched (Fisher's exact test, *P* < 1.89 × 10^−9^) for differentially expressed nodes in the *Gpsm3* DSS knockout signature (forest green) or both (bright green). Nodes present in the CRP, calprotectin, and lactoferrin trait signatures (blue border) are represented. The *GPSM3* subnetwork reflects genes involved in macrophage function. *C1orf228* (encoding p40, the molecular target of ustekinumab) is also present in this subnetwork (red). (**c**) The KDG (diamond) *DOCK2* subnetwork is 2.2-fold enriched (Fisher's exact test, *P* = 0.02) for T cell IBD CRESNPs (light green), 5.13-fold enriched in T cell expression (Fisher's exact test, *P* = 1.84 × 10^−7^), and 4.59-fold enriched for genes upregulated in the *DOCK2* perturbation signature (Fisher's exact test, *P* = 7.91 × 10^−18^) (forest green) or both (bright green). The *DOCK2* subnetwork contains many genes represented in the CRP, calprotectin, and lactoferrin trait signatures from the CERTIFI cohort (blue border). Also represented is the IL-12Rβ1 receptor chain (in red) that comprises a chain in the IL-12 and IL-23 receptor and binds p40, the ligand to ustekinumab. The *DOCK2* subnetwork is also 2.2-fold enriched (Fisher's exact test, *P* = 0.005) in a ROR-γT knockout differential expression signature (triangle). Each circular node represents an expressed gene, and the directed edges connecting genes represent causal or correlative relationships among the genes in the populations from which the network was built.

For each of the IBD networks, the KDGs were all linked via these KDG signatures, as seen in a representative example on the CERTIFI IBD network (Fig. 4a). Furthermore, we identified transcription factors in active regulatory regions of immune cells that have known roles in IBD-associated biology and that are enriched for regulating genes in the KDG subnetworks, suggesting that the KDGs may serve as master regulators of clusters of transcriptional regulators (Supplementary Fig. 7 and Supplementary Tables 29 and 30). Genes in the KDG subnetworks that overlapped with the KDG gene expression signature were also significantly enriched for IBD susceptibility genes that were correlated with clinical traits such as CRP, lactoferrin, and calprotectin in the CERTIFI cohort (Fig. 4b,c and Supplementary Tables 25 and 31).

### *In vivo* validation of intestinal KDGs

**Figure 5:**
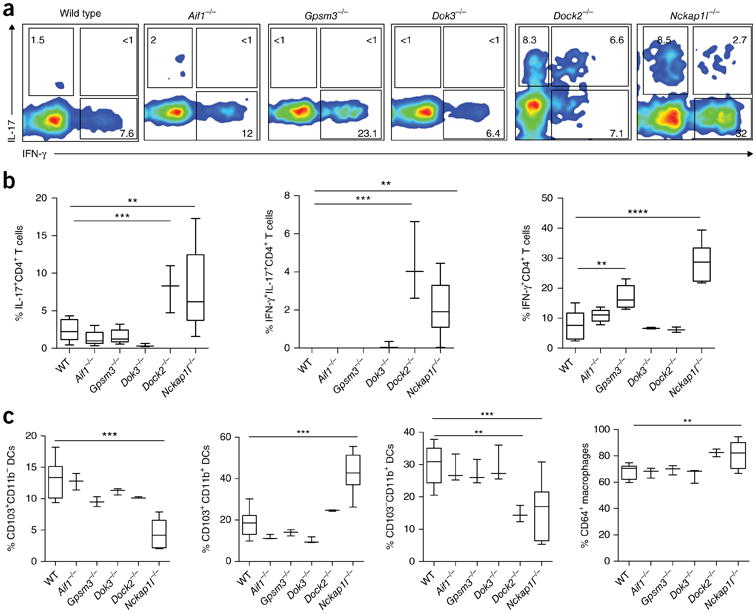
FACS analysis of immune cells in the colonic lamina propria of KDG-knockout mice as compared to wild-type littermate controls. (**a**) Events are electronically gated on CD45^+^CD3^+^CD4^+^ cells, and cells within colored contour plots show staining for IFN-γ and IL-17A. (**b**) Box plots show percentages of CD4^+^ T cells producing IL-17, IL-17 and IFN-γ, or IFN-γ in the KDG-knockout mice. (**c**) Colonic lamina propria cells were isolated from the indicated knockout strains and wild-type controls and stained with anti-CD45, anti-CD11c, anti-CD11b, anti-CD103, anti-CD64, and anti–MHC II antibodies. Cells were electronically gated on CD45^+^CD11^+^MHC II^+^ cells and further subdivided by staining for CD103, CD11b, and CD64. Box plots show the percentages of CD103^+^ DCs, CD103^+^CD11b^+^ DCs, CD103^−^CD11b^+^ DCs, and CD64^+^ macrophages. Data shown are representative of four independent experiments. One-way ANOVA with Bonferroni's multiple comparison was performed. Box limits, first and third quartiles; line, median; whiskers, minimum and maximum values. Statistical significance is indicated as follows: *P < 0.05, **P < 0.01, ***P < 0.001.

To validate the relevance of the intestinal KDGs to IBD, we first examined whether perturbing these genes would disrupt immune homeostasis. For T cells, we evaluated IFN-γ and IL-17A production in CD4^+^ T cells (Fig. 5a,b), as both have been implicated in Crohn's disease pathology^49,50^. *Nckap1l*^−/−^, *Dock2*^−/−^, and *Gpsm3*^−/−^ mice exhibited significant differences in the frequencies of IL-17A^+^ and/or IFN-γ^+^ CD4^+^ T cells in the intestinal lamina propria as compared to wild-type littermate controls. For the myeloid panel, we defined functional subsets of intestinal dendritic cells (DCs) and macrophages using antibodies against CD103, CD11b, and CD64 (refs. 51,52). *Nckap1l*^−/−^ mice had elevated frequencies of CD103^+^CD11b^+^ cells, while both *Nckap1l*^−/−^ and *Dock2*^−/−^ mice exhibited reduced frequencies of CD11b^+^ single-positive DCs and *Nckap1l*^−/−^ mice exhibited reduced CD103^+^ DCs and elevated CD64^+^ macrophages, which could be indicative of an altered IFN-γ or T regulatory response (Fig. 5c)^52^. *Dok3*^−/−^, *Gpsm3*^−/−^, and *Aif1*^−/−^ mice did not have a significantly different immune cell ratio of these myeloid subsets in the colon.

**Figure 6:**
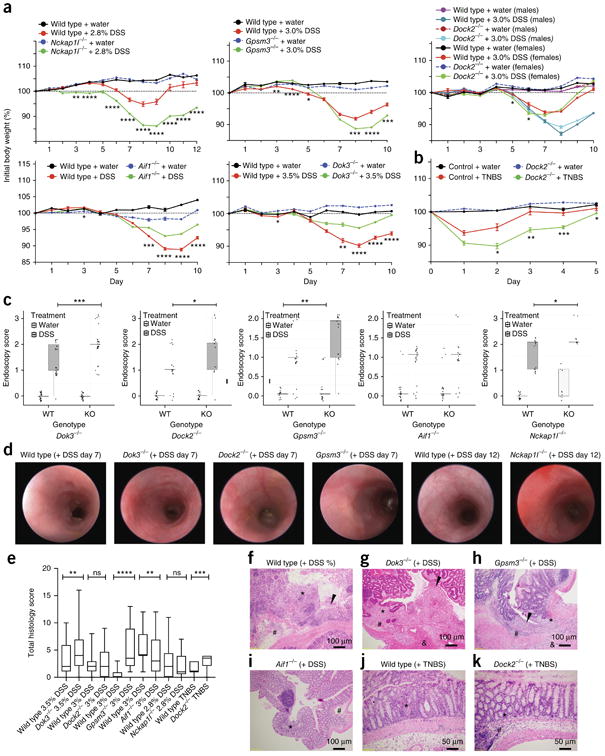
Differential weight loss and intestinal inflammation of KDG-knockout models as compared to sex-matched wild-type littermate controls. (**a,b**) Weight-loss curves for the KDG models. *Nckap1l*^−/−^, *Gpsm3*^−/−^, *Dock2*^−/−^, *Aif-1*^−/−^, and *Dok3*^−/−^ mice under DSS colitis conditions (**a**) and *Dock2*^−/−^ mice under TNBS conditions (**b**) relative to wild-type littermate controls. Comparisons were performed using an autoregressive model to maximize use of the time-series data. (**c**) Colonoscopy severity score on day 7 or 12. Pairwise comparison of endoscopy results was performed using the Mann–Whitney test. (**d**) Images shown are representative of endoscopy scoring performed with blinding to mouse group. (**e**) Histology scores. (**f–k**) Images of sections stained with hematoxylin and eosin. (**f**) A DSS-treated wild-type littermate shows mucosal inflammation, submucosal inflammation, and focal erosion (black arrowhead). (**g**) A DSS-treated *Dok3*^−/−^ mouse shows marked mucosal, submucosal, and muscularis inflammatory infiltrate and ulceration (black arrowhead). (**h**) A DSS-treated *Gpsm3*^−/−^ mouse shows more pronounced inflammation with involvement of the mucosa, submucosa, and muscularis propria and erosion (black arrowhead) as compared to the DSS-treated wild-type mouse. (**i**) Mild mucosal and submucosal inflammation in a DSS-treated *Aif1*^−/−^ mouse. (**j**) A representative TNBS-treated wild-type mouse displaying only focal mucosal inflammation. (**k**) A TNBS-treated *Dock2*^−/−^ mouse shows additional submucosal edema and inflammation as compared to the TNBS-treated wild-type control. *, mucosal inflammation; #, submucosal inflammation; &, muscularis inflammation. Images taken at 10× magnification have a scale bar representing 100 μm, while those taken at 20× magnification have a scale bar representing 50 μm. Data shown represent pooled results from males and females with two independent experiments for each KDG unless otherwise stated. Data are expressed as mean ± s.e.m. Box limits, first and third quartiles; line, median; whiskers, minimum and maximum values; asterisks, significant difference between mice homozygous null for the KDG and wild-type littermate controls treated with DSS: *P < 0.05; **P < 0.01; ***P < 0.001, ****P < 0.0001.

To determine whether the KDGs influenced susceptibility to intestinal inflammation, we employed both innate and adaptive immune mouse models of IBD, including the DSS model and the trinitroben-zenesulphonic acid (TNBS) and T cell (CD45RB^hi^) transfer models of colitis. Wild-type littermate control mice developed signs of intestinal inflammation after treatment (Fig. 6 and Supplementary Figs. 8 and 9). In comparison to littermate controls, *Nckap1l*^−/−^ and *Gpsm3*^−/−^ mice had more severe weight loss and endoscopy scores in response to DSS treatment. *Dock2*^−/−^ mice exhibited significantly greater weight loss in response to treatment with TNBS but less weight loss with DSS treatment.*Aif1*^−/−^ and *Dok3*^−/−^ mice treated with DSS were significantly protected from weight loss (Fig. 6a,b).

We evaluated several parameters of intestinal inflammation in response to colitis in the KDG knockouts: (i) endoscopy score, (ii) histology, (iii) colon weight/length ratio, and (iv) stool score. Homozygotes of the *Dock2*^−/−^ DSS, *Gpsm3*^−/−^ DSS, *Dok3*^−/−^ DSS, and *Nckap1l*^−/−^ DSS mice all exhibited a significantly worse endoscopy score as compared to wild-type littermate controls (Fig. 6c,d). *Dok3*^−/−^ and *Gpsm3*^−/−^ mice exhibited significantly worse histology scores, whereas *Aif1*^−/−^ mice showed a significantly reduced pathology score, as compared to wild-type controls (Fig. 6e-k). In the *Gpsm3*^−/−^and *Dock2*^−/−^ colitis models, the colon weight/length ratio was significantly higher than that in wild-type mice (Supplementary Fig. 8). For stool score, both *Dock2*^−/−^ DSS and TNBS mice, *Dok3*^−/−^ mice, and *Gpsm3*^−/−^ mice presented with a significantly higher score than wild-type mice (Supplementary Fig. 9). To evaluate the contributions of the key regulators in T cell–mediated colitis, we generated the adoptive T cell transfer model for each KDG knockout mouse expected to have altered T cell functions (Supplementary Fig. 10). Results suggested involvement of the KDGs in both T cell and myeloid functions in colitis, with a dominant role in the myeloid compartment; the exception was *Dock2*^−/−^ mice, in which the KDG was demonstrated to have a more dominate role in the T cell compartment. Overall, every KDG-knockout mouse we examined in colitis exhibited a significant weight loss phenotype and an intestinal inflammation phenotype in at least one modality (Table 1 and Supplementary Table 32).

## Discussion

The CIC IBD model validated in this study is unique in having been derived directly from transcriptional variation present in IBD intestinal tissues. By constructing multiple independent network instances of this CIC IBD model across different stages of disease, we were able to independently validate regulatory features conserved across these instances as well as identify conserved patterns of connectivity among them. The premise for our work was to construct a working model of IBD that could in turn be leveraged to identify key drivers of IBD susceptibility genes contributing to active inflammation. The transcriptional profiling of the KDG-knockout mice and the siRNA-mediated knockdowns of KDGs in primary human macrophages validated the network predictions. While our results demonstrate reasonable sensitivity in selecting KDGs (given almost all predictions validated), understanding the specificity is a difficult task. Making formal predictions around genes that do not alter the regulatory states of the network remains a challenging problem, given the familiar saying that absence of evidence is not evidence of absence.

**Figure 7:**
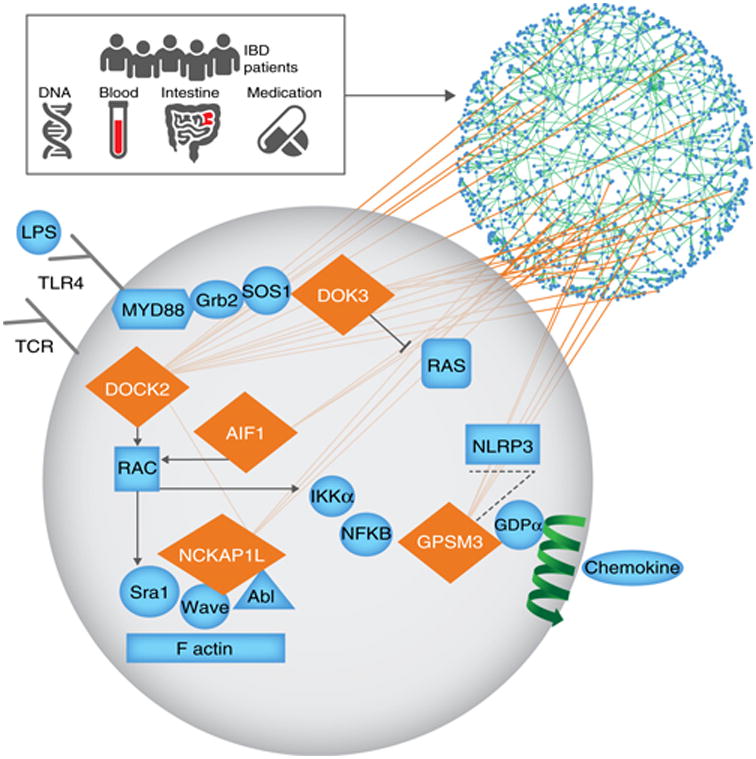
Schematic of crosstalk of KDG molecular and network pathways. The icons represent multiscale datastreams from populations of patients with IBD, including DNA, RNA, and protein collected from blood and intestine of patients across different disease stages. KDG network nodes (diamonds) regulate other network nodes in subnetworks defined by the orange edges, depicting causal regulatory relationships among the network nodes. For example, the NF-κB pathway, RAC, and its actin cytoskeleton rearrangement, RAS, the NLRP3 inflammasome pathways, and TLR and chemokine receptors are modulated by the KDGs we identified, and all have been reported as associated with IBD.

There is support for the mechanistic roles of KDGs in disease being driven by the altered regulatory states of the network resulting from well-defined impacts on processes that are mechanistically associated with immunity (Fig. 7)^24,30,35,53–57^. A unifying theme among the KDGs we identified is RAC activation and cytoskeleton rearrangement, a central mediator in immune processes that has been linked to inflammation in Crohn's disease^58^, experimental colitis^59^, and ulcerative colitis^56,60^. We found significant differences in the myeloid and T cell compartments in ratios of cell subsets at baseline in mouse and in human macrophage function. While there are differences in response between lamina propria–resident macrophages and peripheral blood mononuclear cell (PBMC)-derived macrophages, it is undetermined how these differences compare in homeostasis and inflammatory disease. In our experimental colitis validations with the KDG-knockout mice, all of the knockout models exhibited significantly altered weight loss and an intestinal inflammation phenotype in comparison to wild-type mice. As none of the mutations give rise to spontaneous colitis, these KDGs may not be individually causal but rather may more subtly modulate the regulatory states of the CIC IBD model. The CIC IBD network instances that were enriched for genetic susceptibility and clinical inflammation are disrupted by directed perturbations of the KDGs. Our results demonstrate the high degree of connectivity even among the KDGs, where many of the KDGs had their expression altered by perturbations in other KDGs, highlighting a significant degree of feedback control that will be among the more important refinements that need to be made as the CIC IBD model is evolved.

We believe that the CIC IBD network is the first demonstration of a model constructed from IBD intestinal tissue sourced from three distinct patient populations, providing a unique view into the landscape of disease architecture. The hierarchical organization of the genetic architecture of IBD in a causal network framework, the CIC IBD model, constructed directly from independent populations of patients with IBD, and the identification of the genes that modulate the state of this model, including the discovery of four new regulators of IBD described herein, demonstrate the utility of the CIC IBD model as a resource that others can build upon as the IBD knowledgebase expands. We do not need to have 100% of the genes involved in immune components of IBD in the network for the model to be a useful construct. We just need enough of the pattern to be able to identify the key control points. While differences between specific molecular mechanisms and subsets of disease are of great interest for further exploration of our IBD network, the focus of this work was on identifying and validating master regulators of the CIC network. Identifying the core molecular basis between Crohn's disease and ulcerative colitis at various stages of severity and drug treatment to capture the conserved causal network of susceptibility linked to clinical and pathological inflammation can provide molecular genetic rationale for potentially enrolling patients from both subsets of disease in a single trial for evaluation of a therapeutic target.

### URLs

MSH eQTLs, http://gatkforums.broadinstitute.org/gatk/ discussion/3891/calling-variants-in-rnaseq; RIMBANET (Bayesian network reconstruction), http://research.mssm.edu/integrative-network-biology/Software.html; epigenetic regulatory regions, http://www.ncbi.nlm.nih.gov/geo/roadmap/epigenomics/; visualization for KDG ranking, https://www.synapse.org/#!Synapse:syn7898789; Interactive networks, source data, and source code are publicly available on the Synapse platform at Sage Bionetworks https://www.synapse.org/IBDNetworks.

## Online Methods

### MSH population specimen collection and profiling

Surgical specimens from 134 patients undergoing bowel resection for IBD and non-IBD controls at Mount Sinai Medical Center were collected as the source of tissue. Control samples were collected from normal, non-inflamed bowel located more than 10 cm away from the tumor from patients undergoing bowel resection for sporadic colon cancer. Samples from patients with ulcerative colitis and patients with Crohn's disease were all isolated from areas containing moderate to severe inflammation. The diagnostic pathology report for each specimen was provided by the Mount Sinai Hospital (MSH) Pathology Department. Patients with ulcerative colitis and patients with Crohn's disease had medications in common, including corticosteroids, infliximab, azathioprine, and mesalamine. Samples were collected fresh, and tissue was further processed for isolation. A representative 0.5-cm-wide tissue fragment was isolated from the collected surgical specimen samples, flash frozen, and stored at −80 °C. Tissue was homogenized in TRIzol following the manufacturer's protocol (Life Technologies), and RNA extraction was performed. Specimens with RIN scores >7 were used for poly(A) RNA-seq.

### MSH RNA-seq library preparation and sequencing

About 1 μg of total RNA was used for preparation of the sequencing library using the TruSeq mRNA Seq kit supplied by Illumina (1 FC-122-1001). The protocol followed was according to the manufacturer's instructions. Briefly, mRNA was isolated from total RNA using oligo(dT) on magnetic beads. The mRNA was then fragmented in the presence of divalent cations at 94 °C. The fragmented RNA was converted into double-stranded cDNA. After polishing of the ends of the cDNA, adenine bases were added at the 3′ ends, after which Illumina-supplied specific adaptors were ligated. The adaptor-ligated DNA was amplified by 15-cycle PCR. The PCR DNA was purified on AMPure beads to prepare the final sequencing library. The insert size and DNA concentration of the sequencing library were determined on an Agilent Bioanalyzer. Each RNA-seq library was layered onto one of the eight lanes of an Illumina flow cell at an appropriate concentration and bridge amplified to obtain around 350 million raw reads. The cDNA reads on the flow cell were then sequenced on the HiSeq 2500 platform using a 100-bp single-end protocol. Five barcoded samples were pooled to sequence in one lane. Base calling from images and fluorescence intensities of the reads was done *in situ* on the HiSeq 2500 computer using Illumina software. Various quality control parameters such as the intensities of individual bases and the visual and graphic focus quality of the images were monitored periodically to assess the quality of the ongoing run. Sequence quality was monitored in terms of a colored graphic representation of Q30 values (a measure of errors per 1,000 bases), and error rates at 35 and 75 cycles of sequencing were observed to assess the quality of the ongoing run. The sequencing data generated were simultaneously transferred (in a real-time manner) to a high-performance computer cluster. Short reads from RNA-seq runs were processed and mapped to genes on the basis of the GRCh37/hg19 assembly (UCSC Genome Browser). Short reads in fastQ format were processed using RAPiD, which is a RNA-seq analysis framework developed and maintained by the Technology Development group at the Icahn Institute for Genomics and Multi-scale Biology. RAPiD uses STAR^61^ to map short reads to the [HUMAN: hg19 | MOUSE: mm10] reference, and the resultant alignment map in BAM format is quantified for gene-level expression using featureCounts of the subreads^62^ package. Detailed quality control metrics were generated using the RNASeQC^63^ package. The sequence data were processed for primary analysis to generate quality control values and analyzed using the TopHat and Cufflink pipelines to generate differential expression profiles.

### MSH population eQTL identification

We performed variant calling to identify genetic variants from RNA-seq for eQTL generation^64^ (see URLs).

For each gene-SNP pair, a simple linear regression was used to detect eQTLs

$$y_{i}=\alpha +\beta x_{i}+\varepsilon_{i,1}\leq i\leq n,\varepsilon_{i}\sim N(0,\sigma^{2})$$

where *i* is the subject index, *x* is the effective allele copy number, and *y_i_* is the inverse-normal-transformed gene expression value for subject *i*. The significance of *cis* (SNP within ±1 Mb of the gene location) and *trans* (all others gene–SNP pairs) eQTL effects was quantified with a Wald test on the ordinary least-squares (OLS) estimator of the coefficient *β*. The distribution of the Wald test *P* values under the null hypothesis of no correlation between genotype and gene expression was estimated by rerunning the same analysis on a null data set obtained by permuting the expression sample identifiers. Three permutation rounds were used to construct the null distribution.

### CERTIFI trial

The protocol was approved by the institutional review board at each study center. The study was conducted and reported in accordance with the protocol and statistical analysis plan, available at http://NEJM.org/. All patients provided written informed consent^12^ (Supplementary Table 1).

### Generation of a polygenic risk score for each IBD cohort

An IBD polygenic score^65^ was computed on the basis of a list of IBD-associated SNPs and coefficients^1,2^ and on the basis of 1000 Genomes Project–imputed genotype calls. The list of SNPs used to compute the polygenic risk score was trimmed down to 86 SNPs that were measured in all three cohorts.

### Construction and analysis of coexpression networks

The omental fat coexpression network was generated previously, as described in ref. 8. Coexpression networks were generated for (i) the MSH population: ileum-specific and colon-specific, and ileum and colon combined, coexpression networks; (ii) the CERTIFI network: a pan-intestine network comprising ileum, ascending colon, descending colon, sigmoid colon, and rectum inflamed and non-inflamed tissue; and (iii) an ileum coexpression network from the RISK cohort (see URLs). Additionally, all KDG-knockout and DSS-treated mice were included in the generation of a mouse-specific coexpression network (Supplementary Table 33). See the Supplementary Note.

### The core IAM

We constructed gene coexpression networks for the RISK, CERTIFI, and MSH data sets (Synapse folder) and then calculated the enrichment scores of the resulting coexpression modules for genes in the original immune network^2^. We identified modules across the three cohorts that were statistically significantly enriched for genes in the immune network (*P* < 0.05) and found that they were also highly significantly enriched for immune activation pathways (Supplementary Table 12). The individual coexpression modules comprising the super-immune modules were again among the most enriched for IBD GWAS variants associated with eQTL and/or localized to CREs in immune or digestive tissues. We took the union of genes across these ‘tagged’ modules (referred to as immune super-modules) for each cohort as the most supportive of the immune network (Supplementary Tables 9–11). We purposely employed this less constrained approach to constructing the super-immune modules given that we were seeking to identify a more common set of immune module genes across the networks (see below) and given that this set was to serve simply as a seed set of genes from which to derive a more robust immune-centered IBD network model.

Given our primary aim of constructing a common immune network for IBD across different stages of disease, we identified the most conserved components of the super-modules by taking the intersection across the three IBD populations. This core IAM was dramatically more enriched for the immune network genes, IBD-associated genes, and macrophage-related genes as compared to the individual modules (Supplementary Tables 9–11).

### Reconstruction of the Bayesian networks

The MSH network was generated in the same approach as the CERTIFI and pediatric RISK networks, as previously described^13^.

Bayesian network reconstruction was conducted using the algorithm implemented by RIMBAnet software^66–69^ and visualized by Cytoscape 3.4. The RIMBANET software for constructing Bayesian networks is freely available (see URLs) and comes complete with instructions on how to run the software and specific examples with step-by-step instructions on reproducing previously published results with this software. See the Supplementary Note.

### Identification of disease traits for key driver ranking

We generated the following IBD trait signatures to determine key driver ranking (Supplementary Table 34). See the Supplementary Note.

### Key driver identification

Key driver analysis (KDA) to identify KDGs takes as input a set of genes (*G*) and a directed gene network (*N*; for example, a Bayesian network)^22,23,54,70,71^. The objective is to identify the key regulators for the gene sets with respect to the given network. KDA first generates a subnetwork *NG*, defined as the set of nodes in *N* that are no more than *h* layers away from the nodes in *G*, and then searches the *h*-layer neighborhood (*h* = 1, …, *H*) for each gene in *NG* (HLN_*g*,*h*_) for the optimal h*, such that

$${{\text{ES}}_{h}}^{*}=\max({\text{ES}}_{h,g})\forall g\in N_{g},h\in \{1,\ldots ,H\}$$

where ES*_h,g_* is the computed enrichment statistic for HLN_*g*,*h*_. A node becomes a candidate driver if its HLN is significantly enriched for the nodes in *G*. Candidate drivers without any parent node (i.e., root nodes in directed networks) are designated as global drivers and the rest are local drivers.

#### Macrophage KDGs

The MSG subnetwork was identified with the macrophage-specific gene (MSG) signature. Overlap with the MSG signature was used to identify the macrophage-specific component of the CERTIFI network. Key driver identification was performed by projection of the MSG signature onto the CERTIFI network, the most macrophage-enriched network, and extending out three additional path lengths from the nodes in the network overlapping with the MSG signature. *LAPTM5* was identified as a macrophage KDG through KDG analysis performed by direct projection of the MSS set on the RISK network.

#### Intestine KDGs

We performed KDG identification for the core IAM, extending out two path lengths from the projection of the core IAM on the RISK, CERTIFI, and MSH networks.

### Intestine KDG ranking

The strategy for ranking the KDGs identified in the MSH, CERTIFI, and RISK IBD networks involved assessing the degree to which a KDG was identified in the different IBD signatures that were enriched in our IBD networks. KDGs were ranked in two different ways. See the Supplementary Note for further details.

### Enrichment of variants in CRE regions in cell types and expressed in inflamed and non-inflamed tissue

The significance of the overlap between the gene lists by cell type and anatomical region derived from the CERTIFI patients was assessed using Fisher's exact test, with the full list of causal IBD genes as the background. Once we constructed the immune-cell- and digestive-tissue-specific GWAS signatures described above, we projected them onto networks to identify the largest connected subnetwork associated with each signature. These subnetworks were then tested for enrichment of IBD GWAS genes. See the Supplementary Note.

### KDG transcription factor activity

#### Weight matrices

We downloaded 205 position-specific weight matrices (PWMs) that are supposed to represent individual transcription factors from the JASPAR CORE database^72^. See the Supplementary Note.

#### Enrichment of functional target genes in the subnetwork of KDGs

Inferred transcription factor activity was used to determine the functional target genes for each transcription factor, defined as the genes with the highest total binding affinity for that transcription factor and significant expression correlation with the inferred transcription factor activity. We identified subnetworks for KDGs by searching the neighboring genes (layers 2–4) for each KDG. For each transcription factor and each KDG, the significance level of enrichment for functional target genes and genes in the subnetwork was measured by Fisher's exact test.

### Macrophage KDG-knockdown experimental protocol

#### Differentiation of human monocyte-derived macrophages

Monocytes from three donors were received from Biological Specialty Corporation, and aliquots were frozen using standard procedures. For each experiment, 20 million cells from each donor were thawed, washed with complete medium, and cultured in two T75 flasks with 30 ml of X-VIVO 10 medium (Lonza) supplemented with 10% FBS (Corning), penicillin-streptomycin (Gibco), and 20 ng/ml GM-CSF (R&D Systems). The medium was changed on day 3 and day 7. On day 10, cells were trypsinized and any remaining cells were scraped from the flask. Cells were centrifuged, resuspended in complete medium without GM-CSF, and counted before plating.

#### Macrophage stimulation and siRNA-mediated knockdown

To determine the optimal stimulation conditions for testing network predictions in macrophages, 20,000 cells were plated per well of a 96-well plate and treated with the following stimuli in triplicate wells: 10 ng/ml TNF-α (BioLegend), 20 ng/ml IL-6 (Peprotech), 10 ng/ml IL-1β (eBioscience), or 1 μg/ml LPS. Twenty-four hours after stimulation, cells were lysed in RLT buffer (Qiagen) and processed for RNA isolation and microarray analysis as described below (Supplementary Table 35). For siRNA-mediated knockdown experiments, cells were transfected with siRNAs (Dharmacon) by reverse transfection using Lipofectamine RNAiMAX reagent (Life Technologies). Briefly, cells were plated at 20,000 cells per well in 96-well plates with the indicated siRNA at a final concentration of 10 μM. Three unique siRNAs, each in triplicate, were assayed per targeted gene. Twenty-four hours after transfection, cells were treated with LPS at 1 μg/ml. Twenty-four hours after LPS treatment, the supernatants were transferred to a new 96-well plate and cells were lysed with RLT buffer (Qiagen) + β-mercaptoethanol (Sigma-Aldrich). Lysate and supernatant plates were placed in a −80 °C freezer until they were processed.

#### Cytokine measurement and analysis

To determine the effect of KDG perturbation on cytokine expression, experiments were carried out as above with three separate donors and two independent experiments per donor. Milliplex MAP Human Cytokine/Chemokine Magnetic Bead Panel (38plex from Millipore) assays were set up according to standard procedures using undiluted and 5× diluted supernatants. All out-of-range values were removed, and, for any cytokine, if fewer than 60% of the samples were within range, no analysis was performed. Cytokine response was scaled by dividing over the average for the non-target control by treatment (LPS^+^ or LPS^−^), donor and experiment, or plate. The difference (fold change) in cytokine concentration relative to the non-target control for each siRNA was tested using linear mixed modeling. Fixed effects consisted of a categorical variable for siRNA, and random effects reflected the replicates within each donor and experiment in the first data set and the replicates within each plate in the second.

#### RNA isolation and microarray analysis

RNA was isolated with the Qiagen RNeasy 96-well RNA isolation kit using the standard spin procedure. On-column DNase I digestion was performed, and RNA was eluted using 50 μl of water. A 5-μl aliquot was removed and quantified using the Labchip Pico kit (PerkinElmer). The remaining RNA samples were sent to BioStorage Technologies for microarray analysis using the Axiom U133 Affymetrix GeneTitan Platform. To assess the level of knockdown of each of the targeted macrophage KDGs and the non-target controls, we used the probe sets represented on the Affymetrix microarray used to profile the knockdown experiments that are listed in Supplementary Table 35. Array Studio software (OmicSoft) was used for data analysis. The microarray data were preprocessed and normalized using RMA. Data were log_2_ transformed before analysis to provide normalized intensities. A minimum intensity cutoff of 4.5 was applied to exclude the 40% of probe sets falling below this threshold. A general linear model (GLM) was applied to identify differences in gene expression induced by each stimulation condition.

### Mouse knockout models for KDGs

Male KDG-knockout mice from existing models for *Nckap1l*^17^, *Dock2* (ref. 26), *Dok3* (ref. 42), *Aif1* (ref. 73), and *Gpsm3* (ref. 53) were re-derived at Charles River Laboratories (CRL) with wild-type C57BL6/J female mice purchased from CRL. All mice were crossed heterozygote × heterozygote to maintain litters with mixed genotypes. Mice were co-housed according to age and sex with mixed genotypes (homozygotes, heterozygotes, and wild-type littermates) for a given KDG knockout in a *Helicobacter*- and pathogen-free environment at CRL. See the Supplementary Note.

### Lamina propria immune cell characterization with flow cytometry

Lamina propria lymphocytes were isolated as described^74,75^. Briefly, the intestines devoid of Peyer's patches were incubated in EDTA-supplemented Hank's balanced salt solution (HBSS) without Ca^2+^ and Mg^2+^ (Gibco) for 15–20 min at 37 °C with mild agitation. The epithelial cell layer was removed by vortexing. Remaining sheets of lamina propria were digested in collagenase (Sigma), DNase I (Sigma), and Dispase (BD Biosciences). The cells were resuspended in three 5-ml volumes of 40% Percoll (GE Healthcare) and overlaid onto 5 ml of 80% Percoll in a 15-ml tube. Lymphocytes were collected at the interface of the Percoll gradient, washed once, and resuspended in medium. Flow cytometry experiments were conducted with the following numbers of mice: wild-type littermates, 9; *Aif1*, 5; *Gpsm3*, 5; *Dock2*, 3; *Dok3*, 3; *Nckap1l*, 7. Isolated cells were surface stained in FACS buffer (PBS without Ca^2+^ and Mg^2+^ supplemented with 2% heat-inactivated FBS and 5 mM EDTA) for 20–30 min on ice. Multiparameter analysis was performed on a FACSCanto II (BD), LSR II (BD), or Fortessa (BD), and results were analyzed with FlowJo software (Tree Star). DAPI^+^ cells and doublets were excluded from all analysis. *Ex vivo* stimulations were carried out in the presence of brefeldin A (Sigma), phorbol 12-myristate, 13-acetate (PMA) (Sigma), and ionomycin (Sigma) for 4 h in complete RPMI medium containing 10% FBS (Gibco) at 37 °C. Staining with antibodies to IFN-γ and IL-17A was performed in FACS buffer containing 0.5% saponin (Sigma). DAPI^+^ cells and doublets were excluded from all analysis. Dead cells were excluded using LIVE/DEAD Fixable Violet Dead Cell Stain (Invitrogen) (Supplementary Table 36). See the Supplementary Note.

### Mouse colitis experiments

Mice were used at 8–10 weeks of age. Experiments were carried out using age- and sex-matched groups. The number of mice was determined on the basis of the group size in colitis models required for the models to be well powered to detect a sufficiently large effect size. The numbers of male and female mice used for each KDG colitis model in two independent experiments are given in Supplementary Table 37. See the Supplementary Note.

#### DSS experiments

Colitis was induced by administration of 2.8–3.5% DSS (MP Biomedicals, 10156; molecular weight = 36,000–50,000) to the drinking water from days 0–5. The percentage of DSS was varied depending on the expected susceptibility to DSS-induced colitis, based on the role of the KDG. *Nckap1l*^−/−^ mice were anticipated to be potentially more immune deficient, and a slightly lower dose of 2.8% was therefore used. *Dok3*^−/−^ mice (129sw strain) were more resistant to DSS, and treatment with 3.5% DSS was thus used after treatment with 3% in the first round of experiments. The rest of the experiments were conducted with 3% DSS. Fresh DSS/water solutions were again made on day 3, and any of the remaining original DSS solution was discarded. Beginning on day 5, all animals received fresh filtered water for the remainder of the study. The animals were weighed daily and monitored for signs of distress as well as rectal bleeding. Any animal exhibiting weight loss greater than 30% was killed. Pre-established exclusion criteria included any adverse events and unanticipated deaths, which were reported to the veterinarian immediately. To evaluate colitis severity, animals were anesthetized with isoflurane and subjected to video endoscopy of the lower colon. On days 7 and 10 (days 7 and 12 for *Nckap1l*^−/−^ mice), colitis severity was assessed in all animals using video endoscopy with a small-animal endoscope (Karl Storz Endoskope), where images were taken and colitis severity was scored with blinding to mouse group on days 7 and 10 (or 12) Supplementary Table 38. Mice were killed for histological analysis on day 10 or 12 (*Nckap1l*^−/−^ mice) for DSS^76^.

#### TNBS experiment

The TNBS model was only applied to *Dock2*^−/−^ mice after DSS did not result in a strong phenotype. Colitis was induced by administration of 100 μl of TNBS (4 mg) in 50% ethanol under isoflurane anesthesia on day 0. Colitis was induced by exposure to TNBS, or ethanol for controls, administered intrarectally on day 0. All animals were weighed daily and assessed visually for the presence of diarrhea and/or bloody stool at the time of dosing. On days 3 and 5, colitis severity was assessed in all animals using video endoscopy, where images were taken and colitis severity was scored with blinding to mouse group^76^.

#### Adoptive T cell transfer colitis experiments

The adoptive T cell transfer colitis experiments were carried out as previously described^77^. Briefly, T cells were isolated from the spleens of 8- to 12-week-old knockout and wild-type littermate mice by magnetic cell sorting using the Dynabeads Untouched Mouse CD4 cells kit according to the manufacturer's instructions (Life Technologies). Cells were then sorted on the BD FACS Aria II Cell Sorter (BD Biosciences) at 99% purity for CD4^+^CD25^−^CD45RB^hi^ cells, and 7.5 × 10^5^ cells were injected intraperitoneally into sex-matched *RAG12M* (Taconic) recipients for *Dock2*^−/−^, *Nckap1l*^−/−^, and wild-type littermate control mice and *Rag1^tm1Mom^* (Jackson Laboratory) recipients for *Gpsm3*^−/−^, *Aif1*^−/−^, and wild-type littermate control mice. Mice were weighed weekly until clinical signs of disease were apparent. Antibodies to TCR-β (PerCP-Cy5.5) and CD4 (APC) were used in staining of splenocytes on the day of sacrifice to test for T cell engraftment, which ranged from 3–15% for all mice except *Dock2*^−/−^ mice. Mouse intestine was evaluated at sacrifice for gross anatomical signs of disease.

### Statistical analyses for weight differences in the mouse colitis experiments

Weight loss was a key determinant in assessing the severity of colitis in the mouse models. Given that the experimental design for assessing weight differences between wild-type and knockout animals involved multiple repeated measures over a number of time points for each animal, we leveraged these longitudinal data to enhance the power to detect differences at any given time point using the autoregressive model^78^.

Error bars represent the standard error margin of samples within a group, with *P* < 0.05 indicating significance. The data met the assumption of a normal distribution for all weight and inflammation scores. Variance was measured by s.e.m. The variance between groups was not necessarily similar, but our analysis allowed for differences in variation between groups.

### Statistical analysis for inflammation scores

To assess the statistical significance of differences between knockout and wild-type mice treated with DSS or TNBS, for colon weight/length ratio and stool score, an unpaired two-tailed *t* test was used, and for endoscopy scores (aMann–Whitney test was used). For assessment of the significance for histology, two-way ANOVA and *post hoc* analysis were used, with *P* < 0.05 indicating significance. Significance for flow cytometry was determined using ANOVA and Bonferroni correction and was calculated using PRISM/GraphPad 6.0. Error bars represent the standard error margin of samples within a group.

### Signature enrichments in the Bayesian networks

Fishers exact test was used to evaluate the enrichment of signatures according to a *P* value of <0.05 in the coexpression and Bayesian networks. For Bayesian network enrichment of the mouse colon KDG perturbation signatures, the networks were tested for enrichment and considered significant if Fisher's exact test *P* < 0.05 within the nodes of two path lengths from the KDG within the IBD networks. For the macrophage KDGs, macrophage KDG perturbation signatures with or without LPS were evaluated for enrichment in the networks in which they were identified as described. Signatures enriched with Fisher's exact test *P* < 0.05 were considered significant. See the Supplementary Note.

All human subject research was carried out in accordance with the policies and procedures of Mount Sinai Hospital and its IRB (04-1048 (0002)/HSM 14-00568). The CERTIFI study IRB approval number is CO743T26, and the ClinicalTrials.gov ID is NCT00771667. All patients provided written informed consent.

All animal experimentation was performed in compliance with MSSM (IACUC-2013-1425 PR) and Biomodels (12-1231-2) Institutional Animal Care and Use Committee protocols.

### Data availability

The expression data reported in this paper are available under the following accession codes: Mount Sinai Hospital patient data, GSE83687 (GEO); CERTIFI cohort, GSE100833 (GEO); KDG-knockout mouse intestine expression data, GSE83550 (GEO). All code, data sets, and networks are publicly available on Synapse at Stage Bionetworks (see URLs). A **Life Sciences Reporting Summary** is available.

## Supplementary Material

Supplemental Information

## Figures and Tables

**Table 1:** Summary table of KDG phenotypes

KDG-knockout mouse	KDG expression in IBD patient significantly correlated to CRP, fecal calprotectin, and/or lactoferrin	KDG expression in the intestine correlated with disease duration	Baseline FACS	Endoscopy score	Stool score	Colon weight/length ratio	H&E histology	Colitis model weight loss	Differential cytokine in macrophage KDG knockout	Network validation with enrichment of KDG knockout and knockdown signatures
*Aif1*^−/−^	✓						✓	✓		✓
*Dock2*^−/−^	✓		✓	✓	✓	✓	✓	✓		✓
*Dok3*^−/−^	✓	✓		✓	✓		✓	✓		✓
*Gpsm3*^−/−^	✓		✓	✓	✓	✓	✓	✓	✓ (IL-1RA, CXCL10)	✓
*Nckap1l*^−/−^	✓	✓	✓	✓				✓	✓ (IL-6, CCL4, CXCL10)	✓

H&E, hematoxylin and eosin.
