# Supplementary material for: A functional genomics predictive network model identifies regulators of inflammatory bowel disease
Source: Nat Genet. Author manuscript; Available in PMC 2018 Oct 1. (PMC5660607; doi:10.1038/ng.3947)
Supplement: Supplemental Information [file NIHMS907859-supplement-Supplemental_Information.pdf]

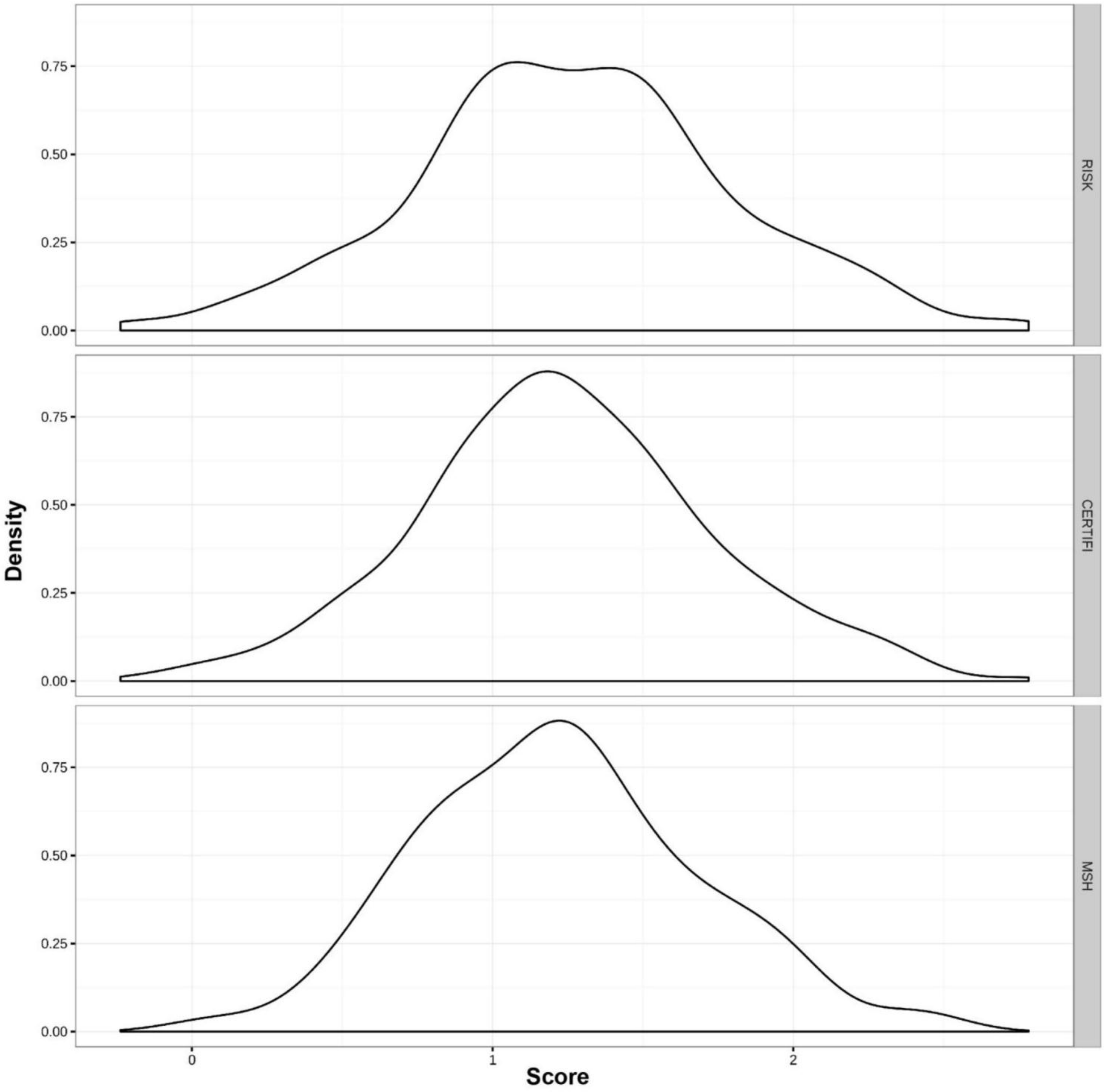

Supplementary Figure 1

Distribution of the IBD polygenic score based on 86 SNPs represented in the three IBD populations (from top to bottom): RISK, CERTIFI, and MSH.

To assess whether the mean polygenic scores for each pair of populations were significantly different, we employed a pairwise *t* test. None of the tests were significant at a nominal 0.05 *P*-value threshold: the *P* value for CERTIFI versus MSH was 0.71; for RISK versus MSH the *P* value was 0.29; and for RISK versus CERTIFI the *P* value was 0.40.

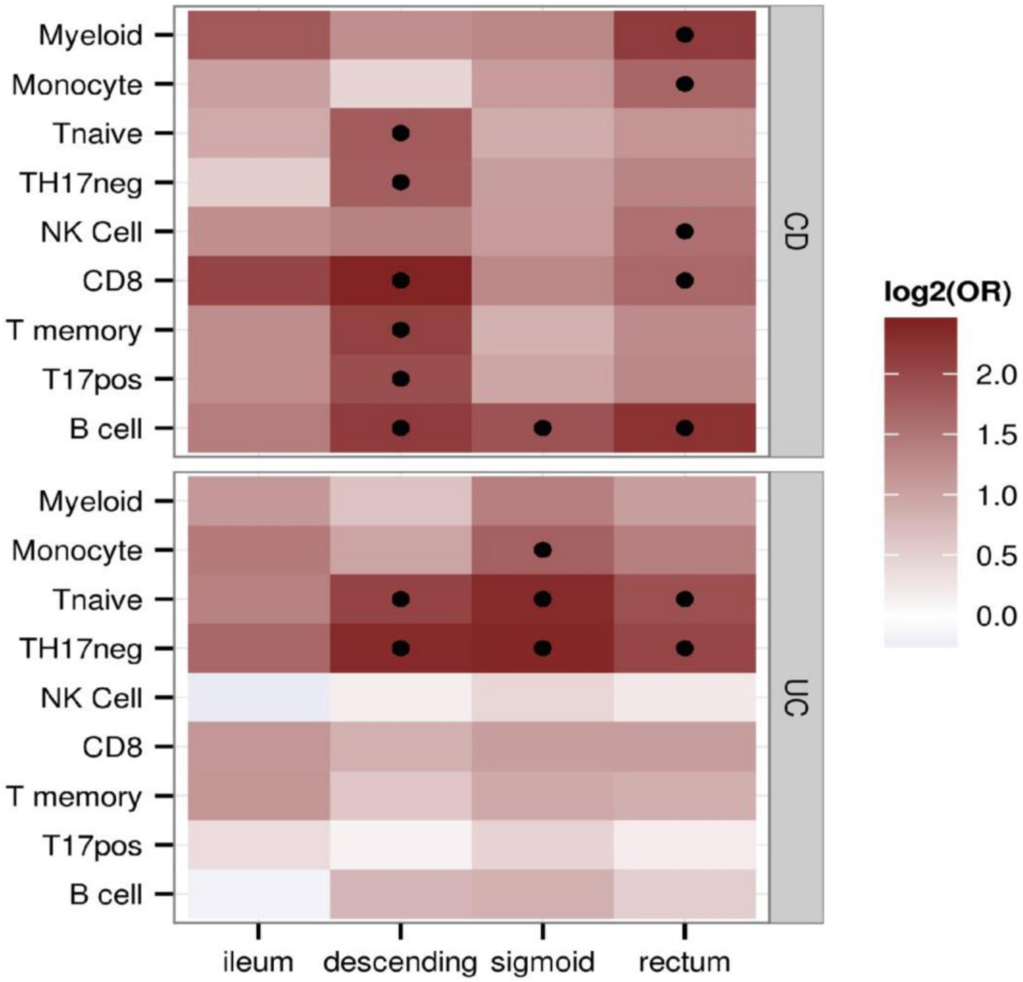

Supplementary Figure 2

Heat map of the odds ratios of overlaps between gene lists by cell type (rows) and inflamed anatomical region (columns) in CD (top) and UC (bottom).

The full list of genes reported in any cell type and/or region was used as the background. Overlaps with Fisher's test $P < 0.001$ are marked with a black circle. Log-odds ratios quantify the overlap between different anatomical regions (in columns) and cell types (in rows) within disease conditions (horizontal panels). We observed, for example, a significant overlap between the myeloid and rectum gene lists within patients with CD but not UC. Included in the heat map is ileum, plus any cell type and anatomical region with at least one overlap with significance, $P < 0.001$.

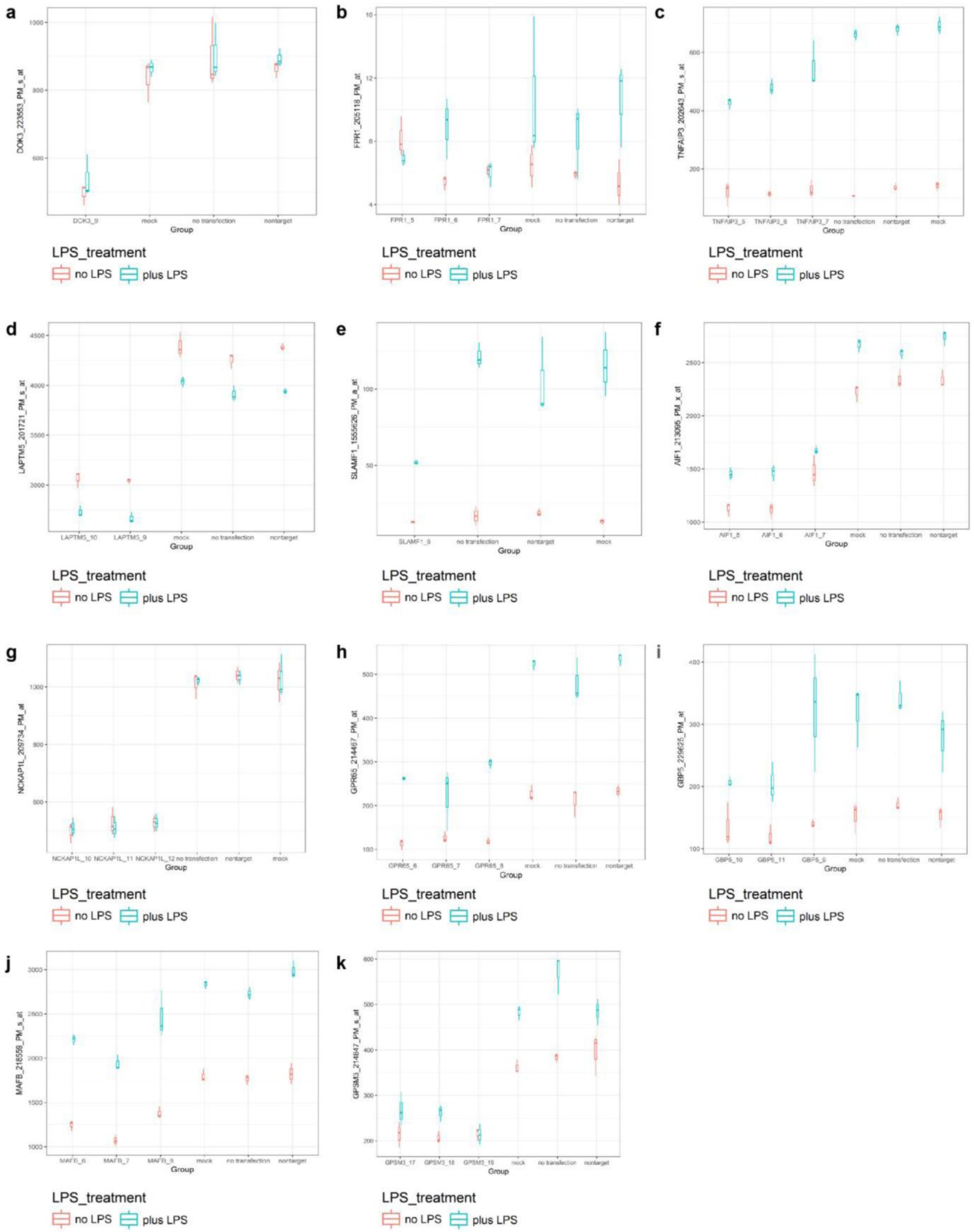
a
b
c
d
e
f
g
h
i
j
k
Group
LPS_treatment
no LPS
plus LPS

Supplementary Figure 3

Extent of KDG mRNA knockdown.

(**a**–**k**) Three siRNA, mock, and non-target control experiments were performed per KDG target, including *DOK3* (**a**), *FPR1* (**b**), *TNFAIP3* (**c**), *LAPTM5* (**d**), *SLAMF1* (**e**), *AIF1* (**f**), *NCKAP1L* (**g**), *GPR65* (**h**), *GBP5* (**i**), *MAFB* (**j**), and *GPSM3* (**k**), in human primary monocyte-derived macrophages with and without LPS stimulation. Data are representative of duplicate or triplicate samples per experiment.

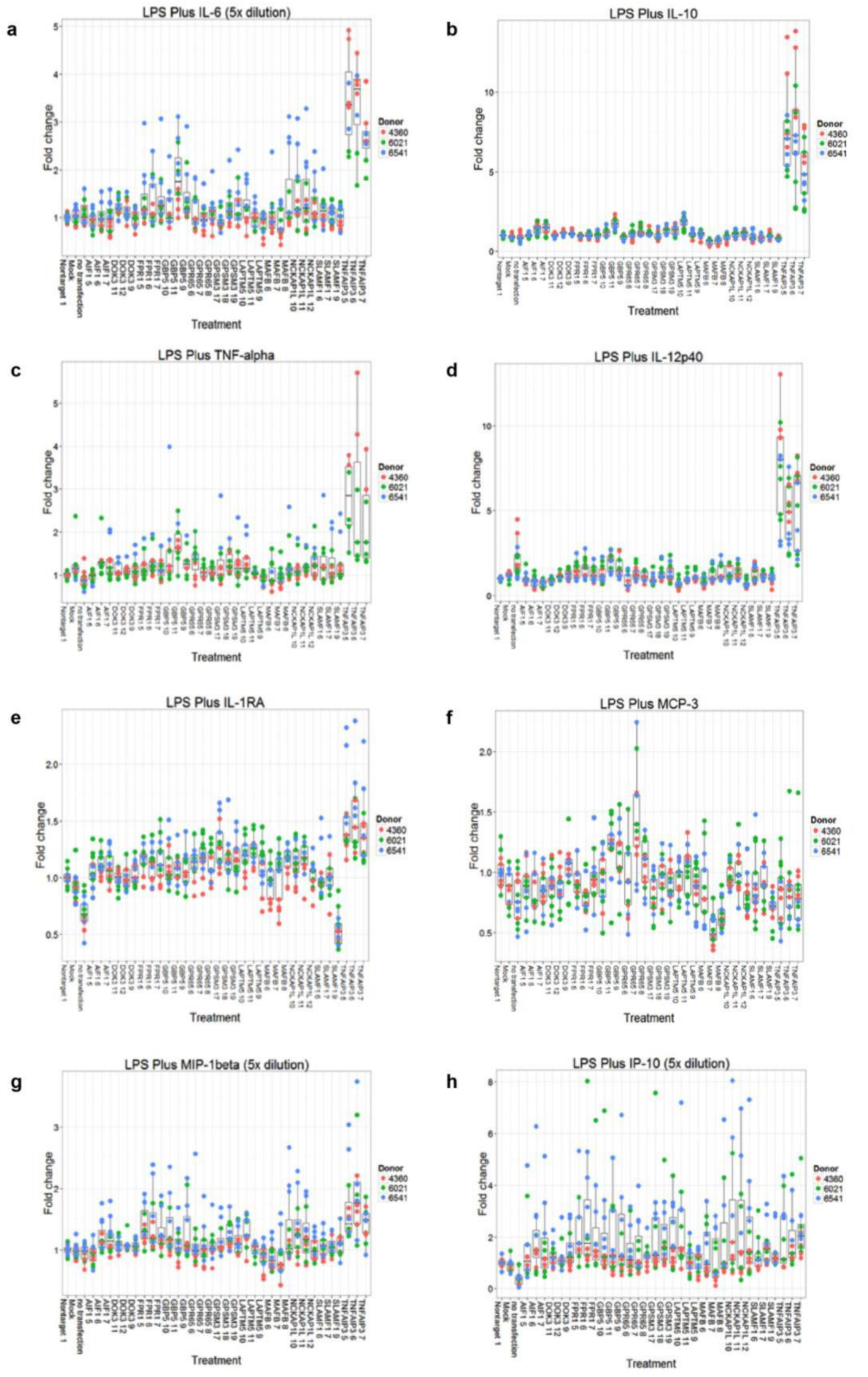
a
LPS Plus IL-6 (5x dilution)
b
LPS Plus IL-10
c
LPS Plus TNF-alpha
d
LPS Plus IL-12p40
e
LPS Plus IL-1RA
f
LPS Plus MCP-3
g
LPS Plus MIP-1beta (5x dilution)
h
LPS Plus IP-10 (5x dilution)
Fold change
Treatment
Donor
4360
6021
6541

Supplementary Figure 4

Effect of KDG knockdown in macrophages on cytokine expression.

(**a**–**h**) Differential production of cytokines following siRNA-mediated knockdown of KDGs: IL-6 (**a**), IL-10 (**b**), TNF-α (**c**), IL-12p40 (**d**), IL-1RA (ILRN) (**e**), MCP-3 (CCL7) (**f**), MIP-1β (CCL4) (**g**), and IP-10 (CXCL10) (**h**). Comparisons were between LPS-treated siRNA versus LPS-treated non-targeting control siRNA cells. Three donors were tested for each siRNA in three separate experiments with two or three replicates per experiment.

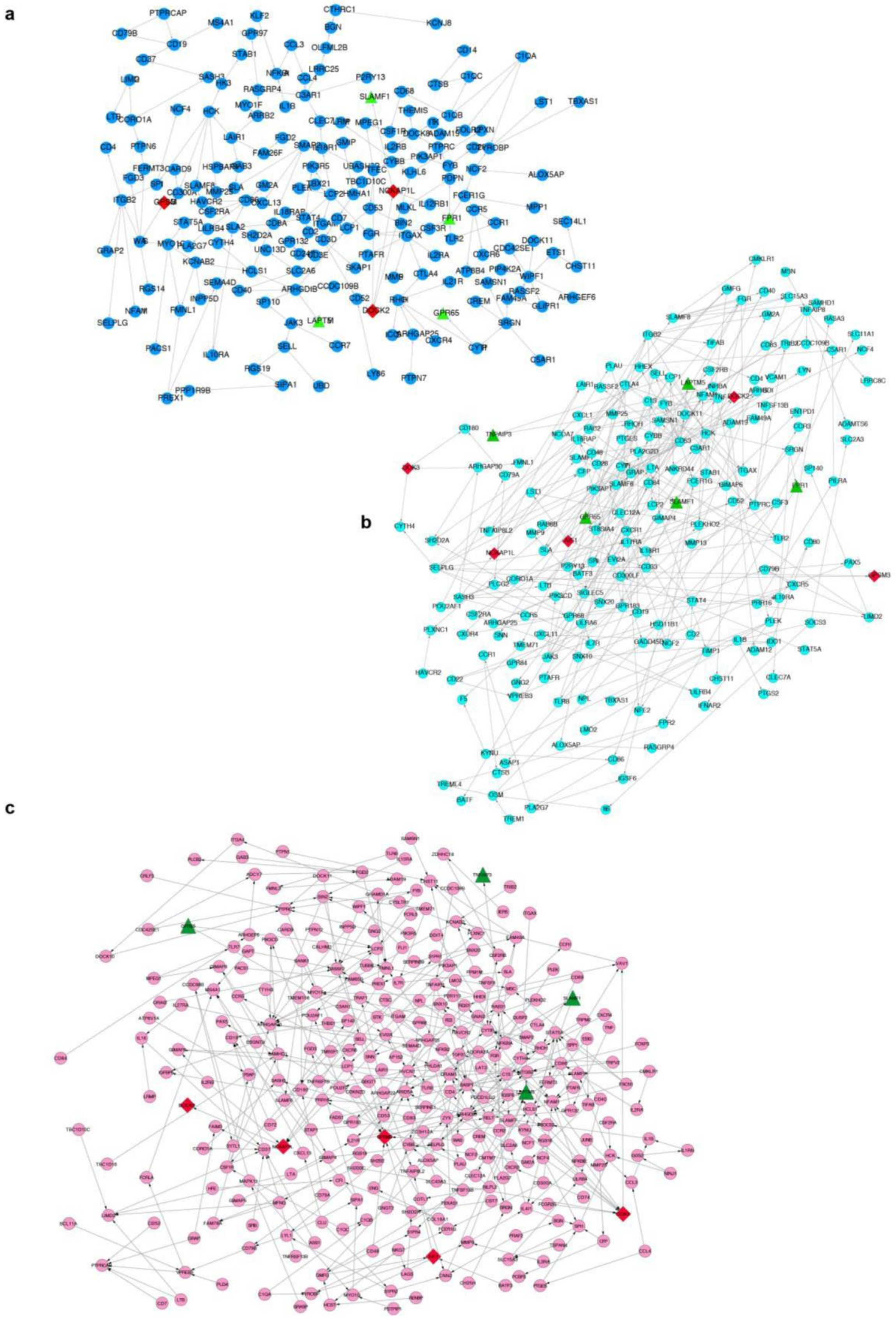

Supplementary Figure 5

The KDG module and network structure are conserved across species.

(**a–c**) Overlap of the mouse brown coexpression module with the CIC subnetwork of the MSH IBD network (184 genes) (**a**), the CERTIFI IBD network (191 genes) (**b**), and the RISK IBD network (308 genes) (**c**). Intestine KDGs are shown as red diamonds. Macrophage KDGs are shown as green triangles.

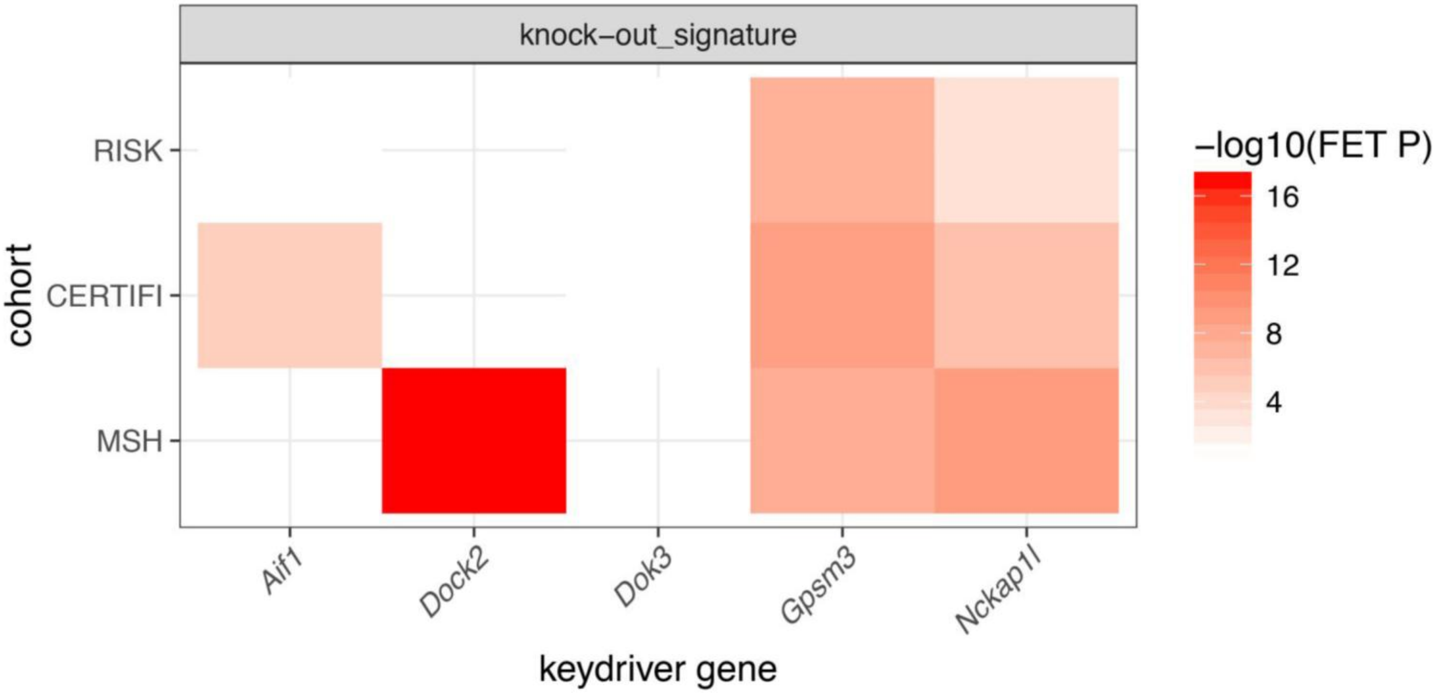

Supplementary Figure 6

Network validation: significant enrichment of KDG perturbation signatures in various IBD Bayesian networks.

Mouse intestine KDG knockout versus wild-type control interaction with DSS differential expression signature $-\log_{10}$ Fisher's exact test *P*-value enrichment within a two-path-length neighborhood of each mouse experimental KDG in the CERTIFI, MSH, and RISK IBD networks.

a

b

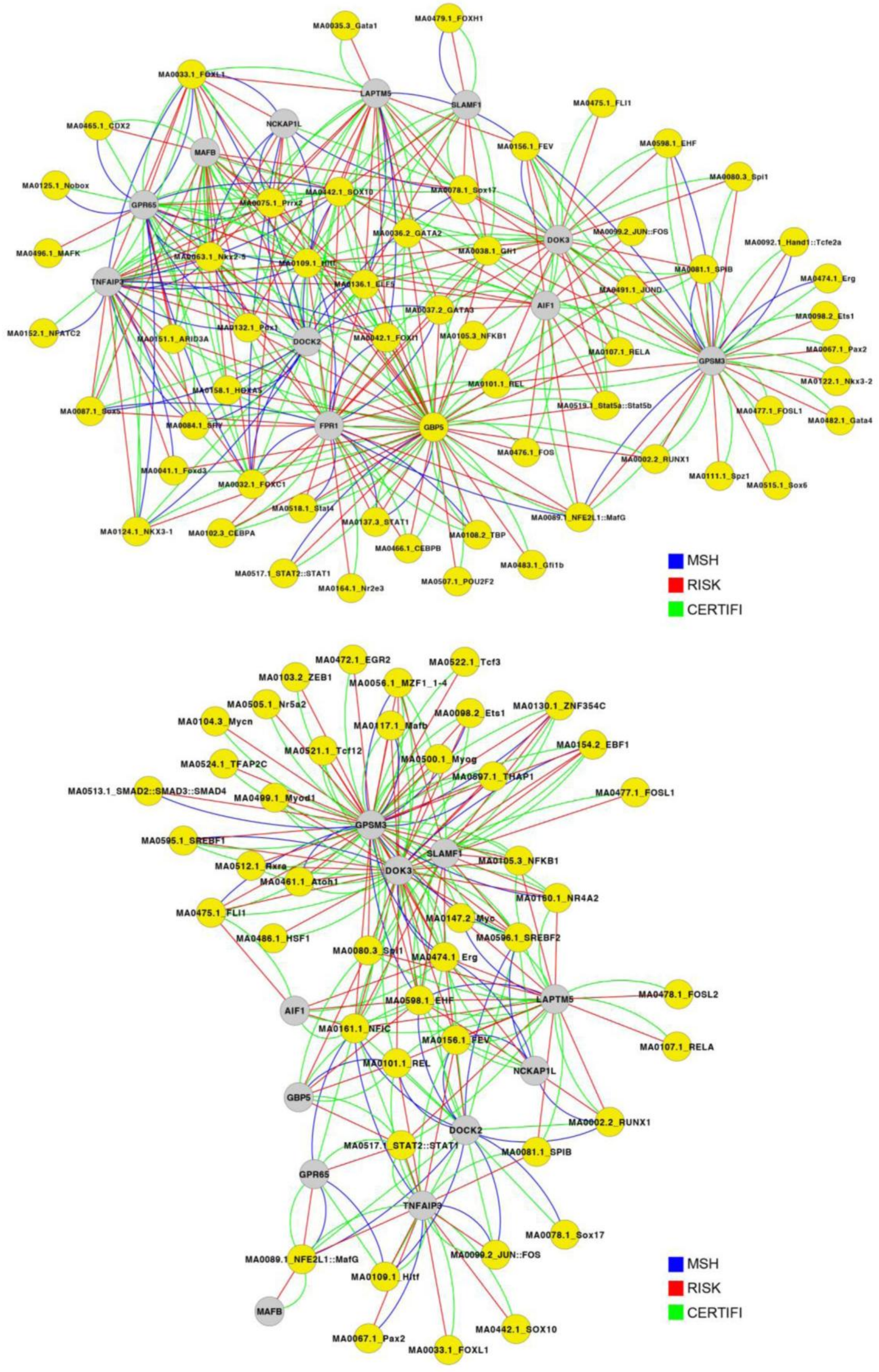

Nature Genetics: doi:10.1038/ng.3947

Supplementary Figure 7

Enrichment of mouse and human transcription factors in KDG subnetworks on each IBD network.

These are transcription factors for regulation of genes in each subnetwork. (**a**) In monocyte results based on DNase I hypersenstivity regions at a significance threshold of $P = 10^{-4}$. (**b**) In T cell ENCODE data at a significance threshold of $P = 10^{-5}$.

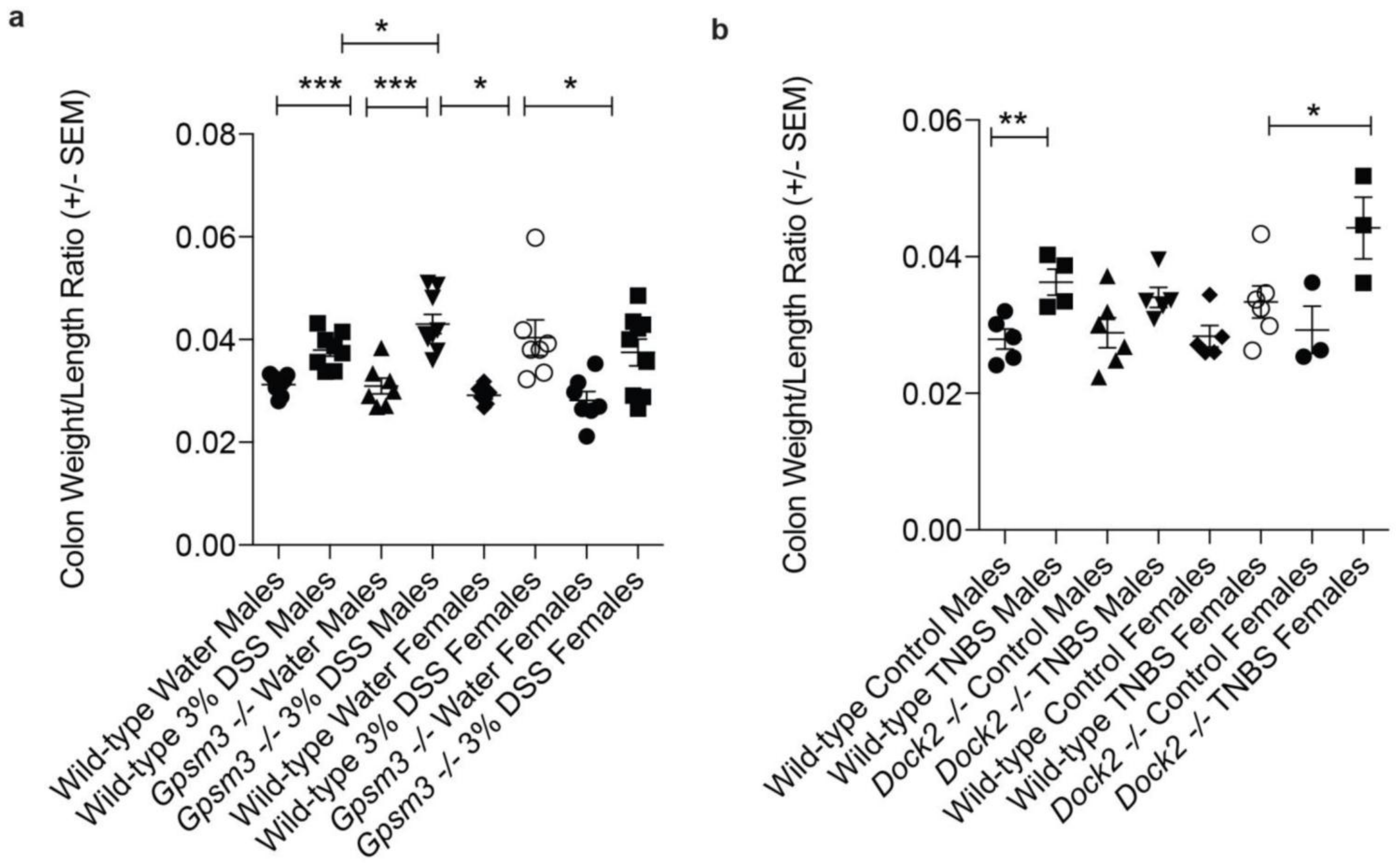

Supplementary Figure 8

Colon weight/length ratio of KDG-knockout mice following treatment.

(**a**) *Gpsm3* DSS: 9 *Gpsm3*$^{-/-}$ and 8 WT male mice. Pooled data representative of two independent experiments are shown. (**b**) *Dock2* TNBS: 3 *Dock2*$^{-/-}$ and 6 WT and female mice. Data are representative of one of two independent experiments. An unpaired *t* test was performed. Data are expressed as ±s.e.m. Statistical significance is indicated: *P < 0.05, **P < 0.01, ***P < 0.001.

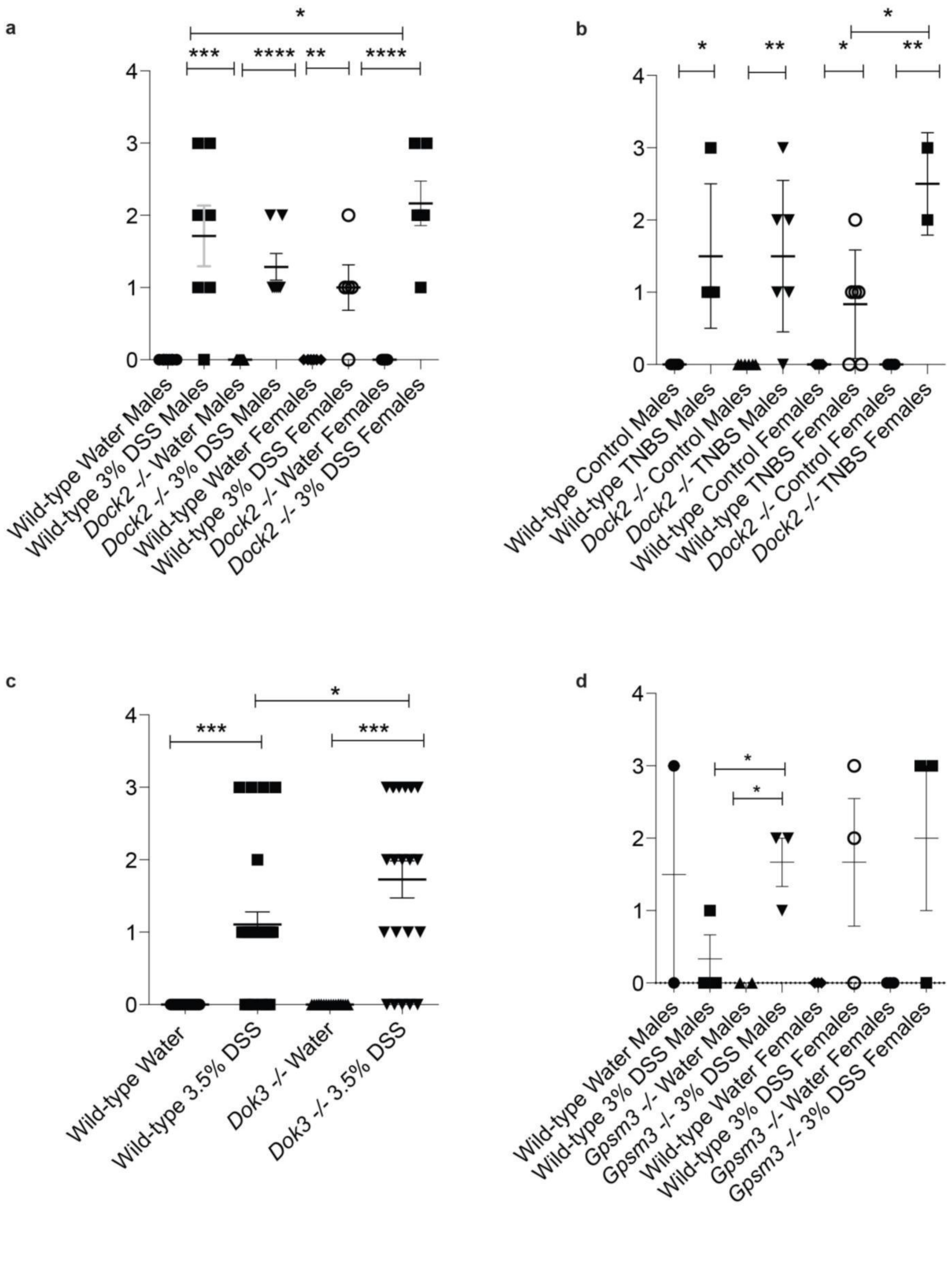
a
Wild-type Water Males
Wild-type 3% DSS Males
Dock2 -/- Water Males
Dock2 -/- 3% DSS Males
Wild-type Water Females
Wild-type 3% DSS Females
Dock2 -/- Water Females
Dock2 -/- 3% DSS Females
b
Wild-type Control Males
Wild-type TNBS Males
Dock2 -/- Control Males
Dock2 -/- TNBS Males
Wild-type Control Females
Wild-type TNBS Females
Dock2 -/- Control Females
Dock2 -/- TNBS Females
c
Wild-type Water
Wild-type 3.5% DSS
Dok3 -/- Water
Dok3 -/- 3.5% DSS
d
Wild-type Water Males
Wild-type 3% DSS Males
Gpsm3 -/- Water Males
Gpsm3 -/- 3% DSS Males
Wild-type Water Females
Wild-type 3% DSS Females
Gpsm3 -/- Water Females
Gpsm3 -/- 3% DSS Females

Supplementary Figure 9

Stool scores from KDG-knockout mice from DSS (day 7) or TNBS.

(**a**) *Dock2* DSS: 6 *Dock2*$^{-/-}$ and 5 WT mice. (**b**) *Dock2* TNBS: 2 *Dock2*$^{-/-}$ and 6 WT mice. The data in **a** and **b** are representative of a single experiment. (**c**) *Dok3* DSS: 20 *Dok3*$^{-/-}$ and 20 WT mice per group. Pooled data representative of two independent experiments are shown. (**d**) *Gpsm3* DSS: 3 *Gpsm3*$^{-/-}$ and 3 WT mice per group. Data are representative of one of two independent experiments. An unpaired two-tailed Student's *t* test was performed. Data are expressed as ±s.e.m. Statistical significance is indicated: $*P < 0.05$, $**P < 0.01$, $***P < 0.001$.

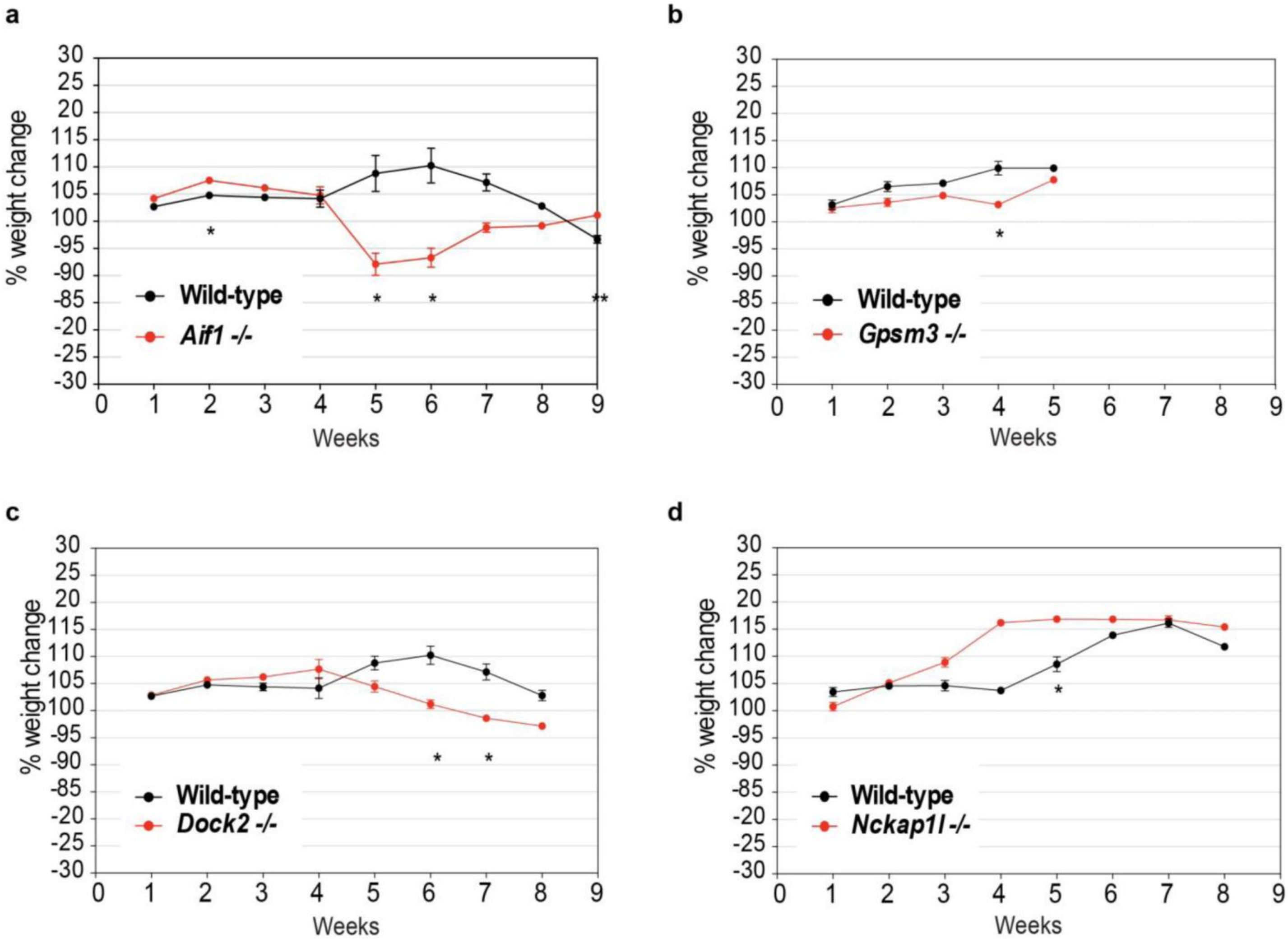

Supplementary Figure 10

Differential weight loss from the T cell adoptive transfer colitis model.

(**a**–**d**) C57BL/6 *Rag*$^{–/–}$ mice were transferred with cells from *Aif1*$^{–/–}$ (**a**), *Gpsm3*$^{–/–}$ (**b**), *Dock2*$^{–/–}$ (**c**), *Nckap1l*$^{–/–}$ (**d**), or respective wild-type littermate control mice. Data are expressed as ±s.e.m. Comparisons were performed using an autoregressive model to maximize use of the time series data. Data are expressed as mean ± s.e.m. *P < 0.05; **P < 0.01; ***P < 0.001.

Supplementary Note

In order to focus on genes linked to IBD, we intersected our eQTL gene list with the GWAS loci identified in IIBDGC and the Immunochip GWAS (See Methods, Supplementary Note) specific to CD and UC. Blood and intestine tissue eQTL were recently reported as enriched for GWAS loci in the CERTIFI population, with roles in autophagy, oxidative stress, microbial sensing by NLRs, the inflammasome, and T cell signaling, as well as for other immune mediated diseases[139]. In addition, we integrated previously published datasets that include IBD-associated eQTL and immune and digestive-related eQTL (Methods, Supplementary Note). DNA variants from these studies significant at the $p < 5e-8$ genome-wide level were intersected with the set of cis-eQTL we identified at a 10% false discovery rate (FDR). In eQTL datasets for which DNA genotype and RNA gene expression data were available, we carried out conditional analysis between top eQTL SNPs and GWAS SNPs to assess whether GWAS SNPs that were also eQTL SNPs were supported as being driven by the same underlying causal variant.

To complement the eQTL data, we curated cell type-specific cis regulatory elements (CRE) across cell types involved in immune and digestive processes using large-scale epigenome study data from the ENCODE, REMC and the Roadmap Epigenomics Project[140], along with other previously published studies[75,141-143]. Given the potential for such regions to modulate the expression of adjacent genes through effects on functional regulatory elements, such as enhancer regions in open chromatin regions[143], we identified all genes with transcription start sites located within 1MB of UC & CD GWAS variants that were also eQTLs in our curated immune and digestive eQTL database and that localized to cell-specific epigenome regions. In order to enrich this set of potential IBD causal genes, we also considered genes proximal to disease-relevant CREs in the absence of eQTL, given the failure of CREs to associate with eQTL may be due to limited power for eQTL detection in existing datasets, expressivity of eQTL in different tissues to which we did not have access, and expressivity of eQTL in unobserved

relevant states or stages of development. For GWAS variants in CREs not associated with an eQTL, CREs were associated with a gene if they were within 5,000 bases of the transcription start/stop site of the gene.

Supplementary Note (Methods)

To build instances of the causal inflammatory component (CIC) IBD model across different disease stages, we employed an integrative genomics approach using this immune network as a seed, as described in Figure 1. The goals of this integrative genomics approach were to a) construct the CIC IBD model and use the model to predict key drivers of IBD pathogenesis, leveraging extensive human data to drive disease understanding[144], (b) validate the physiologic relevance of the key driver genes to IBD using *in vitro* and *in vivo* experimental systems, and c) validate the molecular relevance of the key driver genes to the regulatory states of the model associated with IBD.

To construct a predictive model of the CIC of IBD, we sought to leverage existing knowledge as a seed in our construction process. One representation of this existing knowledge was a molecular network that was significantly enriched for IBD susceptibility loci as well as a wide array of immune pathways such as inflammatory response, innate immune response, defense response to bacterium, T cell activation and interferon-gamma-mediated signaling[62]. Our approach consisted of constructing different representations of the CIC IBD model at different stages of disease.

We constructed three independent, but homologous, probabilistic causal gene networks from intestinal tissues of IBD patients at different stages of the disease (Step c in Figure 1). These CIC IBD networks were then integrated with IBD-related datasets derived from IBD patients, including gene expression signatures derived from disease and inflammation-related contexts and genes in adult and very early onset (VEO) IBD genetic susceptibility loci, to extensively annotate the CIC IBD networks and facilitate identification and prioritization of their master regulators (Steps a, b, c in Figure 1). To identify the master regulators of the CIC IBD networks, we identified those nodes in the networks that were predicted to alter the transcriptional state of a significant proportion of other proximal nodes (referred to as key driver genes, or KDGs). Thus, we prioritized the list of KDGs for experimental validation using a number of criteria that

support their relevance to IBD (Step d in Figure 1). In order to validate the network predictions and explore the role of these predicted KDGs in IBD, we selected 5 top KDGs for prospective *in vivo* experimental and molecular validation in a mouse model for IBD and then an additional 7 KDGs from a macrophage-oriented prioritized list of KDGs for *in vitro* molecular validation in a human macrophage cell system (Step e in Figure 1). The predictive nature of the networks with respect to these KDGs was validated at the molecular level (Step f in Figure 1), and then the impact these KDGs have on IBD biology were further validated for their impact on inflammatory phenotypes in our experimental models (Step g in Figure 1).

Construction of the eQTL/Epigenome Database.

We assembled various datasets in order to annotate genes in a genetic and epigenetic context, and then assembled these datasets into a single database to query for our project. For CD, UC and IBD loci identified in genome-wide association studies (GWAS), we retrieved the International Inflammatory Bowel Disease Genetics Consortium (IIBDGC) and Immunochip (~196,806 SNP CHIP of loci associated with immune disease) studies from http://www.ibdgenetics.org/downloads.html. A combined GWAS/Immunochip replication meta-analysis analysis was performed using both the GWAS and the Immunochip association results comprising 20,700 Crohn's disease cases, 17,865 ulcerative colitis cases and 37,747 healthy controls[62]. Subsequent IBD GWAS data were reported during the course of this study[61], and were integrated into our database. We also included very early onset IBD (VEOIBD) loci in our database[145]. In addition to GWAS loci, we leveraged a number of eSNP datasets to functionally annotate genes in a genetics context to link to IBD genes. This includes a *de novo* set of eSNPs identified from intestinal tissues collected from the MSH IBD population (described above); blood and intestinal eSNPs from the CERTIFI clinical trial population[139]; intestinal eSNPs comprised from ileal biopsy samples of 173 patients undergoing ileal anastamosis[146]; liver, stomach subcutaneous and omental fat eSNPs from a cohort of morbidly obese individuals[147]; T regulatory cell eSNPs identified in 168 donors who are healthy or have type-1 diabetes (T1D) or type-2 diabetes (T2D)[148]; monocyte eSNPs from the studies of Fairfax et al., 2012 [149] and 461,1490, and 432 healthy individuals, respectively[150,151,152]; dendritic cell eSNPs from 534 healthy individuals[151] ; B cell eQTLs[149], blood eSNPs from the KORAF4 study and a study in 5311 individuals [153,154]; brain eSNPs from multiple brain regions [143,155,156] skin eSNPs from the twins study[157]; and then eSNPs for subcutaneous adipose, tibial artery, left ventricle of the heart, lung, skeletal muscle, tibial nerve, thyroid, whole blood and sun exposed skin- all collected from the GTEX study[158]. To complement the GWAS and eSNP datasets, we also

assembled annotations on cis regulatory elements generated on tissues relevant to IBD. ChIP-seq and DHS data generated as part of the ENCODE[141] and REMC projects for human brain/neuron, T-helper cells, liver, skin, and adipose tissue were downloaded from the National Center for Biotechnology Information repository (see URLs). Epigenome annotations that had been generated on intestine[142], monocytes[150], immune cells[140], 9 different cancer cell lines: Nhek, Nhif, Huvec, K562, Hmec, Hsmm, Hesc, Hep92, 2878 [159], and then from multiple other tissues and immune cell types as previously described [160,161] were included. Annotations for enhancers from FANTOM5 and transcription factors from ENCODE were also included. The assembled datasets described above were transformed and standardized such that gene names, genome coordinates, DNA variant identifiers and descriptions were comparable across the datasets. For SNP-based information, genome coordinates were retrieved from the Ensembl database using Biomart. Genome coordinates for all analyses were standardized to hg19 (GRCh37). Cis regulatory elements were represented in BED file format and converted to hg19 genome coordinates from hg18, hg17 using the UCSC lift-over tool. All SNPs and cis regulatory elements were associated with gene symbols (mapping to ENSEMBL gene identifiers) again using the Ensembl Biomart tool. Once data were appropriately transformed and standardized, the data were loaded into a MySQL database. Only eQTLs at a 10% FDR and within 1MB of the transcription start or stop sites of a gene were included. In addition to the regulatory elements we curated as described above, in order to identify genes linked to CD & UC GWAS variants mapped to macrophage enhancer regulatory elements, we used existing annotated epigenome regions[162] and LPS induced eQTLs[91].

GWAS Variant Mapping to IBD Genes

GWAS Positional Annotation.

Variant call format (VCF) genotype files for the European reference sample provided by the November 2010 release of Phase 1 of the 1000 Genomes Project (1000G)[163] were obtained from the 1000G website (http://www.1000genomes.org/). Additional quality control based on standard GWAS procedures was performed on 1000G data using Plink version 1.07[125]. More specifically, individuals were removed if they had missing SNPs > 10%. SNPs were removed if: missing genotype rate > 5%; Hardy–Weinberg equilibrium P value $<10^{-6}$; minor allele frequency <1%. The identity by state and identity by descent analysis implemented by Plink were used for estimation of relatedness. The individual with the higher missingness from each related pair was removed. For each 1000G SNP, the r^2 pairwise LD was calculated within 1,000,000 base pairs (1MB) on either side of the SNP at a threshold of $r^2 \geq 0.8$. We used a mixed approach for

assigning the GWAS SNPs into functional categories similarly to a previous study[143]. For the eSNP functional category, we leveraged the eSNP dataset in the densely mapped 1000G data to identify the GWAS studied SNP that was tagged, as a result of linkage disequilibrium (LD). For generating the CRE or CRESNP functional categories we used a positional approach (ignoring the annotation categories of SNPs in LD with the tag SNP). This mixed model allows us to capture all possible SNPs that affect gene expression (tag or SNPs in LD), followed by positional selection of SNPs that lie within putative regulatory DNA regions.
DNA variants from these studies significant at the $p < 5e-8$ genome-wide level were intersected with the set of cis-eQTL we identified at a 10% false discovery rate (FDR). In eQTL datasets for which DNA genotype and RNA gene expression data were available, we carried out conditional analysis between top eQTL SNPs and GWAS SNPs to assess whether GWAS SNPs that were also eQTL SNPs were supported as being driven by the same underlying causal variant.

To complement the eQTL data, we curated cell type-specific cis regulatory elements (CRE) across cell types involved in immune and digestive processes using large-scale epigenome study data from the ENCODE, REMC and the Roadmap Epigenomics Project, along with other previously published studies[140,159-161]. Given the potential for such regions to modulate the expression of adjacent genes through effects on functional regulatory elements, such as enhancer regions in open chromatin regions, we identified all genes with transcription start sites located within 1MB of UC & CD GWAS variants that were also eQTLs in our curated immune and digestive eQTL database and that localized to cell-specific epigenome regions. In order to enrich this set of potential IBD causal genes, we also considered genes proximal to disease-relevant CREs in the absence of eQTL, given the failure of CREs to associate with eQTL may be due to limited power for eQTL detection in existing datasets, expressivity of eQTL in different tissues to which we did not have access, and expressivity of eQTL in unobserved relevant states or stages of development. For GWAS variants in CREs not associated with an eQTL, CREs were associated with a gene if they were within 5,000 bases of the transcription start/stop site of the gene.

Quantification of GWAS Enrichment in Regulatory Regions.

The quantification of enrichment of GWAS variants for each functional category was done using the categorical enrichment score (CES), as described previously [143,164]. The CES provides a summary score of category-specific enrichment where the mean is taken over all SNP z-scores in the given category. It is estimated based on as the mean $(z^2 - 1)$ and it is justified as a

measure of enrichment based on a simple Bayesian mixture model framework. The CES is a conservative estimate of the variance attributable to non-null SNPs, given a standard normal null distribution and a non-null distribution symmetric around zero. Each empirical null distribution is corrected by applying a control method leveraging only the SNPs without functional annotations (estimated based on SNPs that do not affect gene expression) that are less enriched for true associations. As shown previously[143,164], this approach allows correcting for global variance inflation due to effects of cryptic relatedness and population substructure minimizing the deflation due to over-correction of test statistics for polygenic traits by standard genomic control methods. The inflation factor, λGC was computed as the median z-score squared divided by the expected median of a chi-square distribution with one degree of freedom.

Mapping GWAS Variants to Genes.

In order to identify putative causal IBD genes involved in the regulation of the IBD networks, we mapped CD & UC variants to genes using CRE and eQTL data. First, to identify IBD GWAS variants that are potentially impacting susceptibility by altering the regulation of genes, we mapped GWAS variants to CREs. Then, to associate the CRE with a specific gene (or genes), in cases where the GWAS SNP was associated with an eQTL, we mapped all genes located within 1 MB of the GWAS index eSNP and tested those genes for association to the expression of each gene identified using the eQTL datasets. Association between gene expression and SNP genotype was carried out using linear regression, after adjusting for age, race, and gender. If the GWAS index SNP was associated with expression of the gene of interest, we identified all eSNPs for the corresponding gene that were in LD with the GWAS index SNP and then carried out conditional analysis on the eSNPs and GWAS index SNP to provide support for the eQTL and IBD locus being driven by the same underlying causal variant. In cases where the CRE-associated GWAS index SNP was not associated with the expression of any proximal gene, we assigned genes based on the proximity of the CRE regions harboring GWAS index SNPs to the structural gene. Specifically, to define the regulatory regions, if a CRE region of interest was overlapping or contained within a gene region (defined by the minimal transcription start and maximal transcription stop site annotated for the gene) or within 5000 bases of the transcription start or stop sites, it was then associated with that gene. We annotated whether the CRE region was: 1) located before the 5' end of gene but within 5000 bases, 2) at the 5' end of gene, spanning transcription start site, 3) within the gene, 4) spanning the entire gene, 5) at the 3' end of gene, spanning transcription stop site 6) after the 3' end of gene but within offset bases.

Identification of Causal Variants.

The functional datasets used herein were divided into three groups: eSNP (eQTL), cis regulatory elements (CREs), and CRE eSNP (eSNPs falling within CREs). For each eSNP dataset, those SNPs within 1MB of the transcriptional start or stop sites of a gene and significantly associated with the expression of that gene ($p < 10^{-4}$) were considered as cis eSNPs. To associate cis eSNPs with GWAS variants, we identified GWAS variants that were significant at a genome-wide significance level of 10^{-8}, and then overlapped these variants with the cis eSNPs. It was our aim to identify cis-eQTL in our curated immune and digestive database that was coincident with the IBD GWAS signal. For those eQTL datasets for which we had both DNA and RNA, we carried out a conditional analysis as previously described [165] to assess whether GWAS variant and top cis eSNPs were independent or likely driven by the same causal variant. This analysis was performed as an annotation and did not affect the mapping to gene results. (1) We looked for interesting cis-eQTL signals (unadjusted P of IBD SNP association with transcript expression $<1.0x10^{-4}$) for transcripts that mapped within 1 MB of the lead IBD GWAS SNPs and were expressed in >5% of the samples. (2) For each transcript with an interesting cis-eQTL, we checked whether there was a nearby SNP with a more convincing association with expression of that transcript (transcript peak SNP). If the IBD GWAS signal and the cis eQTL were coincident, one would expect the P for the IBD SNP association with the transcript expression to be equally small as the P for the transcript peak SNP. (3) If there was a transcript peak SNP different from the cis eQTL SNP, but in high LD ($r^2>0.7$) with the IBD GWAS SNP, this was another indication for the IBD GWAS signal and the cis-eQTL signal being coincident. (4) We performed mutual conditional analyses computing the association of the IBD GWAS SNP adjusted for the transcript peak SNP and vice versa. If the association of the transcript peak SNP disappeared (P>0.05) by adjusting on the IBD GWAS SNP, this was the final indication for the IBD GWAS signal and the cis-eQTL signal being coincident, i.e. that the IBD GWAS SNP signal was mediated through the respective gene and that the transcript was probably implicated in the cis- eQTL modulation.

Genetic Signatures.

Signatures included the following:

Genes established as very early onset IBD genes in genetic studies[145]. A GWAS signature generated by taking the union of all genes associated with CD & UC GWAS variants which are associated with an eQTL and are located in a cis regulatory element (CRE) AND GWAS

variants mapped to genes proximal to location in a CRE. Cell type enrichment in CRE regions annotated in REMC and ENCODE Roadmap include the union of genes mapped to CRE regions in: digestive tissues, CD8 T cells, CD4 T cells, NK cells, B cells and myeloid cells including monocytes, dendritic cells, neutrophils.

Clinical Variable and Pathology Correlation Signatures.

Spearman correlation analysis was conducted under the R working environment for clinical variables & normalized intestine gene expression at Week 0 at baseline for Fecal Calprotectin Lactoferrin, CRP, and disease duration data collected in the CERTIFI trial. Signatures were generated by identifying all genes expressed in the intestine with a Rho correlation of: +/- 0.3 (since fecal calprotectin (850 genes) and lactoferrin (481 genes) had a stronger correlation with gene expression in the intestine than CRP (695 genes) and disease duration (342 genes), for which a threshold of +/- 0.2 was used in order to capture all relevant genes for network analysis. For the absolute Rho, i.e., the |Rho|, criteria as follows: 0.00-0.19 “very weak”, 0.20-0.39 “weak”, 0.40-0.59 “moderate”, 0.60-0.79 “strong” 0.80-1.0 “very strong”. MSH patient intestine samples were scored on a scale of 1 to 3 according to disease severity (1 = non inflamed, 2 = moderate inflammation, and 3 = severe inflammation) according to the pathology report. A gene correlation signature was then generated using this disease severity score and the MSH gene expression data using Kendall's correlation according to a threshold of FDR < 0.02 and P< 0.0005.

Differential Expression Signatures.

A differential expression signature of colonic CL vs CD and CD vs UC and ileal CL vs CD derived from the MSH population restricted to fold change, FDR <0.05 and the latter P< .05 as well as differential expression signatures of: CD inflamed vs non inflamed ileum CD inflamed vs non inflamed descending colon, CD inflamed vs non inflamed sigmoid and CD inflamed vs non inflamed rectum from the CERTIFI trial restricted to an FDR< 0.01. See Computing IBD Patient Gene Expression Signatures.

Macrophage Signatures.

The Macrophage Specific Signature (MSS) was generated from deriving the union of the following signatures: 1) Co-expression modules from human macrophages were sourced from the M1, M2 and TPP (TNFα, PGE, and TPP- (TLR2 ligand P3C)) stimulated macrophages[166] 2), a TB infected human DC signature[62], and 3) human macrophages treated with 100nM of

Dexamethasone[167]. This MSS signature was also used to identify enrichment of macrophage expressed genes in addition to T cell expression[168] in the 11 macrophage Key Driver Gene (KDG) sub network.

We complemented the MSS with an independent macrophage-specific set of genes, macrophage specific genes (MSG), that we identified by carrying out experiments to determine what genes appear to be specific to macrophage expression (across a broad range of macrophage contexts). We isolated monocytes from human blood and transformed them into monocytes from 600 individuals and carried out RNAseq on those samples[169]. In addition, we isolated microglial cells, astrocytes, and neurons from mouse brains and RNA sequenced those individual cell types as well. We then identified genes that were observed as expressed in any of the human macrophages or mouse microglial cells, but not expressed in the astrocytes, neurons, nor in any of the M induce pluripotent stem cell lines we had previously established[170]. This produced a set of 500 genes that were considered as the MSG set.

Computing IBD Patient Gene Expression Signatures.

The IBD patient signatures include:

1) For MSH differential expression analysis, we used SAMseq[171] and DESeq[172] to perform differential expression analysis on read counts of RNA-seq data collected from intestinal biopsy samples. Briefly, raw read counts were normalized to account for differing sequencing depths of each sample, and a non-parametric model was used to identify differential transcripts between two-class comparisons. Permutation testing was performed (n = 1000) to identify transcripts with significant expression differences between groups and we corrected for multiple hypothesis testing using q-values, setting the false discovery rate (FDR) at FDR < 5%. Most of the differentially expressed transcripts have q-values < 1%. To identify a molecular signature for IBD (CD and UC), we performed differential expression analysis on RNAseq data of processed tissue samples collected from cases and controls undergoing surgery. The model to test for transcriptional differences included age and gender as covariates. While we observed thousands of differences between cases and controls or hundreds of differences for the direct comparison between IBD cases (CD vs. UC), almost no differences were seen for age, and only a few dozen sex-specific transcripts differed for gender. We calculated expression-level differences between cases and controls while including age and gender as covariates for ileum CD vs CL, colon CD vs CL, colon UC vs CL, colon CD vs UC. For each tabular file, (provided on Synapse- see URL), we have summary statistics for each mapped transcript (total of 23,228). The baseMean mean transcript expression across all samples was taken and the $\log_2$ fold-

change (ratio of X_vs_Y, e.g. $\log_2(Y=X)$) lfcSE standard error estimate for log fold-change. Stat is the statistical score output from negative binomial test.

2) For the CERTIFI trial differential expression analysis, whole-genome transcriptional profiles were measured from biopsy tissue samples collected from individuals with CD who participated in the CERTIFI trial in part as previously published[73]. To ascertain tissue-specific signatures, we examined inflamed versus non-inflamed tissue from various anatomical regions of the small intestine, colon, and rectum. For array processing, a total of 321 biopsy tissue samples were collected from 86 individuals at Week 0 of the study in addition to samples taken at other time points in the study. For each participant, 4 samples were collected on average from different anatomical regions of the terminal ileum, colon, and rectum. Expression profiles were measured on the Affymetrix Human Genome U133 Plus 2.0 Perfect Match platform (GPL570), which contains 54,715 probes that map to 21,095 unique Entrez gene IDs. Pre-processing of expression data included background correction, $\log_2$ transformation, quantile normalization, and Robust Multi-array Averaging (RMA) to stabilize the variance and rescale the levels across arrays. Array differential expression results summary: normalization was performed with the R package affy. Further quality control measures of clustering and principal component analysis (PCA) were employed to identify potential outliers in the data. Age and gender adjustments to expression levels were performed on each tissue location using robust linear regression with the R package limma[173]. Only samples with valid age and gender data were used for analysis. Expression data was preprocessed using RMA, separately for blood (N=150) and intestine samples. When not available, gender was inferred by information on expression of the Y linked gene RPSY41. Subsequently, gene expression values were adjusted for age, gender, tissue, clinical site and the top 5 principal components (derived from the gene expression data itself) in a robust linear model to accommodate potential outliers for expression level, separately for blood and intestine data to whole-genome expression data collected from biopsy tissue samples and whole blood of individuals in the CERTIFI trial. To identify tissue-specific differences between inflamed and non inflamed biopsies samples, replicate samples from the same individual were removed and two-class unpaired analyses were applied between groups using the Significance Analysis of Microarray (SAM) algorithm[171] implemented by the siggenes package in R. Briefly, SAM calculates a statistic based on the relative difference in gene expression values between groups, $\frac{d(i) = \overline{x}_I(i) - \overline{x}_u(i)}{s(i) + s_0}$ where $\overline{x}_I(i)$ and $\overline{x}_u(i)$ are the average expression levels in the inflamed and non-inflamed tissues for the ith probe. The denominator

reflects the probe-specific standard deviation across all samples $s(i)$ plus small positive constant chosen to minimize the coefficient of variation and to help ensure the variance of $d(i)$ is independent of gene expression. P values are estimated by permutating sample labels and calculating an empirical score distribution to compare with the score between the actual groups. The family-wise error rate was controlled by calculating probe-level q-values. To control for multiple hypothesis testing, we used the Benjamini & Hochberg adjustment on the raw P values to control the family-wise error rate and set a false discovery rate threshold of 1% or 5%. All analyses were performed using the R statistical package, version 2.15.24.1.

Mouse Colon KDG Knock out Differential Expression Signatures.

RNA sequencing data was normalized by RNA-seq count data being transformed into $\log_2$ counts-per-million units and each gene was assigned an observational weight according to the voom-transformation [174,175]. These weights were used to adjust for count heteroscedasticity across the biological replicates by incorporating the mean-variance trend within the R statistical programming package limma pipeline[175] which allowed gene expression to be modeled as a linear function of the experimental design for differential expression. Differential expression analysis was carried out between the control and homozygous knock out mice groups (genotype groups), the DSS and control groups (treatment groups), and the interaction between the genotype and treatment groups (the DSS interaction signature), using the lmFit and eBayes functions in the LIMMA package. Across each of these contrasts, we computed differential expression at the gene (DEG) level for the mm10 Ensembl build. Multiple testing across all genes identified in the RNAseq data was accounted for by controlling the family-wise error rate using permutation methods to empirically estimate the null distribution of no difference between any of the two groups tested. From the permuted data, a P value threshold was selected to control the false discovery rate[176]. We generated a single signature for each KDG KO, looking for genotypic effect including interaction with the condition (e.g., DSS treated or not), and using condition as the baseline to compare against. A 5% FDR was used to select the genes for each signature.

Macrophage siRNA Knock down Differential Expression Signatures.

We examined a primary human macrophage cell system under different stimulation conditions, including stimulation with TNFα, IL6 and LPS, to identify a stimulus that impacted expression of KDGs and their neighboring genes. Lipopolysaccharide (LPS) was used as a macrophage activation treatment. Eleven key driver genes, including *AIF1*, *DOK3*, *FPR1*, *GBP5*, *GPR65*,

GPSM3, *LAPTM5*, *MAFB*, *NCKAPIL*, *SLAMF1* and *TNFAIP3*, were knocked down with siRNA in human primary macrophages. Twenty-eight distinct gene perturbation experiments and three types of controls were performed. The experiments were performed in triplicates totaling 186 samples with gene expression data profiled with the Affymetrix HT HG-U133_Plus_PM Plate array (Supplementary Table 35) (GEO platform, GPL13158). Microarray raw data were normalized with “rma” of the “affy” BioC package[177]. To remove non-expressed genes, probes were filtered with the threshold of 50, resulting 27803 probes. Differential gene expression analysis was carried out with the “limma” package [172,173]. Due to relative small sample sizes per group (n=3), FDR criteria were not applied. Instead, three thresholds were employed for identification of differential expression genes, including 1.2, 1.5, and 2.0 fold change compared to the non-target controls (P<0.05). Given each gene targeted by a few distinct siRNAs in these experiments, in order to the increase the statistical power, further analyses were also carried out, in which transcriptomic data from siRNA with similar efficiency were combined. Putative statistically significant probes were mapped to HGNC symbol based on the latest annotation of GPL13158 platform for the downstream gene signature analysis.

Signature Enrichment in Bayesian Networks

In order to identify gene perturbation signatures, we identified up-regulated (denoted as UP) and down-regulated (denoted as DN) genes by applying a composite threshold requiring expression fold change > 1.2 and nominal t-test P value < 0.05 for macrophage knock-down experiments. The UP and DN signatures were combined to further generate “ALL” signatures to account for simultaneous positive and negative regulations in gene regulatory networks. For the mouse intestine perturbation signatures, we looked for genotypic effect including interaction with the condition (e.g., DSS treated or not), and using condition as the baseline to compare against and then a 5% FDR was used to select the genes for each signature. These signatures were then subject to enrichment analysis in l-layer network neighborhoods (l = 1,...,lmax, where lmax= 2) expanding from the key driver gene perturbed in the respective experiment in each full Bayesian Network (BN) signature and macrophage specific component of the CERTIFI network depending on how the KDG was identified. The enrichment statistics were evaluated via Fisher’s Exact Test (FET) within each BN, by taking the vertex set of global BN as the background genes. The sub network in Figure 3c is defined by projecting the cytokines differentially expressed in the knockdown of key driver *TNFAIP3* in the macrophage onto the CERTIFI IBD network and extending out two path lengths of nodes to identify the largest connected sub graph.

Cell enrichments from co-expression networks.

Cell Expression Enrichment Mapping of GPL570 probesets to cell types was downloaded from The Gene Expression Barcode 3.0 [178]. Enrichment analysis was performed to retrieve the cell types most strongly associated with a queried list of genes using the SaddleSum tool [179].

Co-expression Network Module Enrichment.

To determine the enrichment of a gene set from a module or differential expression signature in the network, we calculated the fold-change and P value. Fold-change enrichment (E) was calculated as a ratio of ratios E = (a/b)/(c/d) where a is the number of IBD-associated genes in the module of interest, b is the number of IBD-associated genes, c is the total number of genes in the module of interest, and d is the total number of genes in the full co-expression network. This can also be applied to enrichment in Bayesian sub networks of interest as compared to the full Bayesian network. We used the hypergeometric distribution to calculate the P value and assess the significance of each enrichment calculation. For the IBD CRE SNP union signature enrichment in the RISK, CERTIFI, and MSH modules as well as the enrichment of the RISK, CERTIFI and MSH modules in the immune activation network modules, enrichment was ranked by P value and a P value threshold of <0.01 was applied.

Pathway Enrichment.

Pathway enrichment using KEGG and GO was performed on the RISK, CERTIFI, MSH and KDG mouse co-expression modules using the Molecular Signatures Database. We tested each of these co-expression modules for enrichment of Gene Ontology (GO) categories, KEGG, Panther, and GeneGo pathways through Fisher's exact test corrected for multiple testing.

Construction and Analysis of Co-expression Networks.

Weighted gene co-expression network analysis begins with a matrix of Pearson correlations between all gene pairs in the dataset of interest, then converts the correlation matrix into an adjacency matrix using a power function $f(x)=x^\beta$. The parameter β of the power function is determined in such a way that the resulting adjacency matrix (i.e., the weighted co-expression network) is approximately scale-free. To measure how well a network satisfies a scale-free topology, we use the fitting index[180] (i.e., the model fitting index R^2 of the linear model that regresses log(p(k)) on log(k) where k is connectivity and p(k) is the frequency distribution of

connectivity). The fitting index of a perfect scale-free network is 1. For this dataset, we select the smallest β which leads to an approximately scale free network. The distribution of the resulting network approximates a power law: $p(k) \sim k^{-y}$. To explore the modular structures of the co-expression network, the adjacency matrix is further transformed into a topological overlap matrix[180]. As the topological overlap between two genes reflects not only their direct interaction, but also their indirect interactions through all the other genes in the network, previous studies[180,181] have shown that topological overlap leads to more cohesive and biologically meaningful modules. To identify modules of highly co-regulated genes, we used average linkage hierarchical clustering to group genes based on the topological overlap of their connectivity, followed by a dynamic cut-tree algorithm to dynamically cut clustering dendrogram branches into gene modules[182]. To distinguish between modules, each module was assigned a unique color identifier, with the remaining, poorly connected genes colored grey.

Reconstruction of the Bayesian Networks.

In brief: Bayesian networks are directed acyclic graphs in which the edges of the graph are defined by conditional probabilities that characterize the distribution of states of each node given the state of its parents. The network topology defines a partitioned joint probability distribution over all nodes in a network, such that the probability distribution of states of a node depends only on the states of its parent nodes: formally, a joint probability distribution $p(X) = \Pi i$ on a set of nodes X can be decomposed as $p(X) = \prod_i p(X^i | Pa(X^i))$, where $\mathrm{Pa}(X^i)$ represents the parent set of X^i. In our networks, each node represents transcription expression of a gene. These conditional probabilities reflect not only relationships between genes, but also the stochastic nature of these relationships, as well as noise in the data used to reconstruct the network.

Bayes formula allows us to determine the likelihood of a network model M given observed data D as a function of our prior belief that the model is correct and the probability of the observed data given the model: $P(M|D) \propto P(D|M) * P(M)$. The number of possible network structures grows super-exponentially with the number of nodes, so an exhaustive search of all possible structures to find the one best supported by the data is not feasible, even for a relatively small number of nodes. We employed Monte Carlo Markov Chain (MCMC) simulation to identify potentially thousands of different plausible networks, which are then combined to obtain a consensus

network (see below). Each reconstruction begins with a null network. Small random changes are then made to the network by flipping, adding, or deleting individual edges, ultimately accepting those changes that lead to an overall improvement in the fit of the network to the data. We assess whether a change improves the network model using the Bayesian Information Criterion (BIC)(Schwartz), which avoids over-fitting by imposing a cost on the addition of new parameters. This is equivalent to imposing a lower prior probability $P(M)$ on models with larger numbers of parameters.

Even though edges in Bayesian networks are directed, we can't infer causal relationships from the structure directly in general. For example, in a network with two nodes, X^1 and X^2, the two models $X^1 \to X^2$ and $X^2 \to X^1$ have equal probability distributions as $p(X^1, X^2) = p(X^2 | X^1)p(X^1) = p(X^1 | X^2)p(X^2)$. Thus, by data itself, we can't infer whether X^1 is causal to X^2, or vise versa. In a more general case, a network with three nodes $X^1, X^2,$ and X^3, there are multiple groups of structures that are mathematically equivalent. For example, the following three different models, $M1: X^1 \to X^2, X^2 \to X^3$, $M2: X^2 \to X^1, X^2 \to X^3$ and $M3: X^2 \to X^1, X^3 \to X^2$, are Markov equivalent (which means that they all encode for the same conditional independent relationships). In the above case, all three structures encode the same conditional independent relationship, $X^1 \wedge X^3 | X^2$, X^1 and X^3 are independent conditioning on X^2, and they are mathematically equal

$$p(X) = p(M1|D) = p(X^2|X^1)p(X^1)p(X^3|X^2)$$
$$= p(M2|D) = p(X^1|X^2)p(X^2)p(X^3|X^2)$$
$$= p(M3|D) = p(X^2|X^3)p(X^3)p(X^1|X^2)$$

Thus, we can't infer whether X^1 is causal to X^2 or vise versa from these types of structures. However, there is a class of structures, V-shape structure (e.g.

$\text{Mv}: X^1 \to X^2, X^3 \to X^2$),

which has no Markov equivalent structure. In this case, we can infer causal relationships. There are more parameters to estimate in the Mv model than M1, M2, or M3, which means a large penalty in BIC score for the Mv model. In practice, a large sample size is needed to differentiate the Mv model from the M1, M2, or M3 models.

Incorporating genetic data as a structure prior in the Bayesian network reconstruction process. In general, Bayesian networks can only be solved to Markov equivalent structures, so that it is often not possible to determine the causal direction of a link between two nodes even through Bayesian networks are directed graphs. However, the Bayesian network reconstruction algorithm can take advantage of the experimental design by incorporating genetic data to break the symmetry among nodes in the network that lead to Markov equivalent structures, thereby providing a way to infer causal directions in the network in an unambiguous fashion[126]. We modified the reconstruction algorithm to incorporate eSNP data as prior as following: genes with cis-eSNP[183] are allowed to be parent nodes of genes without cis-eSNPs, but genes without cis-eSNPs are not allowed to be parents of genes with cis-eSNPs, $\mathrm{p}(trans \rightarrow cis) = 0$. We have shown that integrating genetic data such as cis-acting eSNP or eQTLs (excluding edges into certain nodes) improves the quality of the network reconstruction by simulations [127] and by experimental validations [127,128]. We note that in applying this particular version of the Bayesian network reconstruction algorithm (incorporating genetic information as a prior), if genetic information is not available or is ignored, the population is simply treated as a population with random genetic perturbations.

Averaging network models. Searching optimal BN structures given a dataset is an NP-hard problem. We employed an MCMC method to do local search of optimal structures as described above. As the method is stochastic, the resulting structure will be different for each run. In our process, 1,000 BNs were reconstructed using different random seeds to start the stochastic reconstruction process. From the resulting set of 1,000 networks generated by this process, edges that appeared in greater than 30% of the networks were used to define a consensus network. A 30% cutoff threshold for edge inclusion was based on our simulation study[127], where a 30% cutoff yields the best tradeoff between recall rate and precision. The consensus network resulting from the averaging process may not be a BN (a directed acyclic graph). To ensure the consensus network structure is a directed acyclic graph, edges in this consensus network were removed if and only if (1) the edge was involved in a loop, and (2) the edge was the most weakly supported of all edges making up the loop.

The computational complexity of our MCMC method for described above is, $\mathrm{O}(N^4)$ where N is the number of nodes included in the network reconstruction process. It is practically impossible to construct a global Bayesian network including all 39,000 genes. Therefore we used variance

of gene expression as a threshold, taking the top 25% of most expressed genes in the tissue. Following the procedure described above, 1,000 BNs were reconstructed using different random seeds to start the reconstruction process. From the resulting set of 1,000 networks generated by this process, edges that appeared in greater than 30% of the networks were used to define a consensus network. Our previous simulation study shows that the 30% inclusion threshold results in a stable structure and achieves the best tradeoff between precision and recall[127]. The histogram of percentage of occurrences of all potential edges shows that 30% is a reasonable cutoff threshold for inclusion.

Intestine KDG Ranking

For the first ranking approach, we ranked the KDGs with respect to genes associated with IBD GWAS, VEOIBD, CRP, CD vs. CL ileum, disease duration, CD vs. CL colon, CD vs. UC colon, CD UC gwas/eqtl/epigenome consensus, and the original immune IBD network. For each KDG the signature of that KDG was computed by identifying all genes that are predicted to be altered by that gene within a path length of 2. This KDG signature is computed with respect to each CIC network. The KDG signature was then intersected with each of the trait signatures (trait gene sets) and a Fisher exact test was applied to assess whether the overlap was more than would be expected by chance. Thus, for each KDG a P value (or alternatively considered, a fold-enrichment) is derived that can then be used to rank order each KDG from most significant to least for each given trait in each CIC network. Thus for each trait and each CIC IBD network, you get a ranking from 1 to N for the N KDGs under consideration. Given the number of KDG varies between networks, the ranks are normalized by dividing through by N. The median across all traits was then computed. After rank ordering the KDGs for each trait in each network, the mean of the ranks across all of the lists across all networks was computed, collapsing all of the trait-associated lists down to one rank ordered list of KDGs (column C in the supplementary table 16, where the ranks were normalized by dividing through by the highest rank).

The second ranking approach involved ordering the KDGs across the CIC IBD networks by counting the number of times a KDG signature (as defined above) was enriched for the different trait signatures (as defined above). The KDGs were rank ordered based on this count, the motivation being that KDGs that were more consistently observed as having the potential to alter genes associated with different IBD traits, were more likely to be common across the networks. The normalized ranks from this process are provided in column D of supplementary table 15. For the two rank-ordered lists constructed, the mean of the ranks was computed and

these mean rank values were in turn rank-ordered to produce the final ranking used to prioritize KDGs for experimental validation (column A). Thus the score we constructed was based on the significance of the impact the KDG was predicted to have on its local network neighborhood within the CIC IBD network across all trait signatures considered, as well as the number of times the KDG signature was significantly enriched in the IBD disease trait signatures (see Figure 2 and Supplementary Table 16 for the rankings based on each trait considered).

Visualization for Key Driver Gene Ranking.
The pipeline for plotting circular track diagram is now implemented as a R package NetWeaver. You can find a brief description and example usage about this package from Synapse (see URLs).

A login (free registration if you don't have an account) is required to see the example image in this link.

TF binding affinity profiles. We used the *convert2psam* utility from REDUCE Suite version 2.0 software package (bussemakerlab.org) to convert each PWM from JASPAR to a position-specific affinity matrix or PSAM [184,185]; pseudo-counts equal to one were added to the PWM at each position, and the resulting base counts were divided by that of the most frequent base at each position to get an estimate for the relative affinity associated with each point mutation away from the optimal binding sequence. The resulting PSAM collection was used to compute a promoter affinity for each gene. All putative individual binding sites in the genomic region from 10kb upstream to 10kb downstream of the TSS of each gene with a predicted relative affinity of at least 0.1 were identified and scored using the *AffinityProfile* utility in the REDUCE Suite. The individual binding sites are summed to calculate total binding affinity of TF f binding to a gene g as follows: $K_{fg} = \sum_{i \in U_g} K_{fgi}$, where K_{fgi} represents the binding affinity between TF f and the DNA at position *i* within cis-regulatory sequence U_g. We obtained genome sequence for *Homo sapiens* from R Bioconductor package BSgenome.Hsapiens.UCSC.hg19. We further consider tissue specific accessible DNA for TF binding. In particular, we used DNase I hypersensitivity regions, which correspond to tissue-specific accessible DNA for TF binding[185]. We calculate TF binding affinities by: $K_{fg} = \sum_{i \in U_g^{DnaseI}} K_{fgi}$, where U_g^{DnaseI} represents DNase I hypersensitive regions

Nature Genetics: doi:10.1038/ng.3947

U_g^{DnaseI} among the cis-regulatory sequence

U_g.

Inferring TF activity. The TF binding affinities can be used to infer sample specific TF regulatory activity by performing genome-wide linear regression with genome-wide mRNA expression profile [186,187]. For each sample, linear regression of the genomewide mRNA expression on the total promoter affinity for each TF was performed as follows: $y^s = b_0^s + \sum_f b_f^S K_f + e$, where y^s represents mRNA expression of sample s, a

K_f !

represents a total promoter affinity for one of TF f. The regression coefficients of the total promoter affinity b_f^s were interpreted as sample-specific TF activities. We performed this procedure with different sizes of cis-regulatory sequence, 1kb through 10kb of upstream and downstream sequences, then selected optimal sizes of cis-regulatory sequence by enrichment between genes with high total binding affinity and genes whose expression level is correlated with TF activities. The inferred TF activity with the selected optimal sizes of cis-regulatory sequence was used further analysis.

Mice in colitis experiments

Mice were delivered from Charles River Labs and acclimatized 1-2 weeks before experimentation. The study was performed in animal rooms provided with HEPA filtered air at a temperature of 70 ± 5°F and 50% ± 20% relative humidity. Animal rooms were set to maintain a minimum of 12 to 15 air changes per hour. The room was on an automatic timer for a light/dark cycle of 12 hours on and 12 hours off with no twilight. Animals were fed with a sterile Purina Labdiet® 5053 rodent diet and sterilized water was provided *ad libitum*. In each cohort, male or female mice of the same strain were randomized into twelve (12) total groups of six to ten (6-10) animals unless numbers were otherwise stated. Each animal was identified by an ear punch corresponding to an individual number.

Statistical Analyses for Weight Differences in the Murine Colitis Experiments

In particular, if we let y_{tl} represent the weight change measure for animal l at time point t, then our autoregressive model of interest is:

$y_{tl} = \gamma_{0t} + \gamma_{lt}\theta_l + \sum_{j=1}^{t} \phi_{tj}(y_{t-j,l} - \hat{y}_{t-j,l}) + w_{tl}$ for $t = 1, 2, ..., k$ (the k time points over which weight measures were taken), where $\mathrm{var}(w_{tl}) = \sigma_t^2$ for $l = 1, ..., n$ and n is the number of animals in the study. Here θ_1, is the genotype indicator for the gene of interest, taking the value 0 for wild type animals and 1 for KO animals. $y_{t-j,l} - \hat{y}_{t-j,l}$ represents the weight measures at time point $t - j + 1$, where $\hat{y}_{t-j,l}$ is the prediction of $y_{t-j,l}$ from the previous model. In taking this difference as the weight measure for a time point of interest, the effect of genotype on weight at previous time points is effectively removed. After fitting the model at the desired time point, the parameter γ_{lt} can be tested for significance using a standard t test. Therefore, the null hypothesis for the test is that there is no difference in weight between the KO and WT groups, conditional on genotypic effects from the previous time points.

Scoring of Phenotypes from Colitis Experiments.

Colitis was scored visually on a 5 point scale that ranges from 0 for normal, to 4 for severe ulceration. Each mouse was assigned a single score that corresponded to the most severe damage observed throughout the entire length of the colon according to 1) the Endoscopy Crohn's Scoring Scale for DSS and TNBS and the 2) Stool Consistency Scoring Scale for DSS & TNBS (Supplementary Table 38).

3) Histology Scoring for DSS & TNBS: Slides were scored in a blinded manner by a gastrointestinal pathologist according to the severity of colitis using the following histological parameters: mucosal involvement- normal (0) 3-10 mucosal neutrophils/ hpf (1), more than 10 mucosal neutrophils or rare crypt abscesses (2), multiple crypt abscesses or erosion (3); submucosal involvement- normal (0), focal aggregates of neutrophils (1), neutrophil infiltration with expansion of submucosa (2), diffuse neutrophil infiltration (3); muscularis- normal (0), scattered neutrophils within the muscularis (1), neutrophil infiltration with focal effacement of the muscularis (2), extensive neutrophil infiltration with transmural effacement of the muscularis (3).

In addition, we scored crypt damage as follows: normal (0), loss of the basal one third (1), loss of the basal two thirds (2), entire crypt loss (3), crypt loss with surface erosion (4) and confluent, extensive erosion (5). For evaluation of ulcers, the absence of ulceration was scored (0), one to two ulcer foci (1), three to four ulcers foci (2), confluent extensive ulceration (3). The total score per mouse was evaluated for statistical significance[188].

Collection of Biological Material from the Colitis Experiments and Profiling

1) Stool was collected and evaluated directly from colon at sacrifice. The presence of diarrhea and/or blood in the stool were assessed visually and scored. Upon sacrifice, the colon was excised, the contents were removed and collected, and its length and weight were measured. The colon was then trimmed to a length of 5 cm and divided into three pieces. The distal 2 cm were placed in RNA Later (Ambion) for subsequent isolation of RNA, while the middle 1 cm portion was placed in formalin for subsequent histopathology examination. Samples stored in RNA Later were stored at 4°C for 24 hours and then transferred to -20°C for long-term storage. The 1 cm piece of transverse colon isolated from each mouse in the DSS experiments for histology was placed between two sponges in a cassette and fixed in 10% phosphate-buffered formalin before processing in paraffin-embedded blocks. Four-micrometer sections were cut at the mucosa level and stained with hematoxylin and eosin. Slides were viewed using an Olympus BX43 microscope and images were captured with an Olympus DP26 camera and Olympus cellSens image acquisition software.

2) RNAseq of the mouse colon: The miRNEasy 96 kit was used for isolation of the mouse distal colon samples. DSS treated and untreated homozygote and wild type distal colon samples from the DSS experiment with RIN>6 were included for further study with at least 8-10 samples per group for expression analysis. RNA sequencing libraries were prepared using the KAPA Stranded RNA-Seq with RiboErase sample preparation kit (kapabiosystems) in accordance with the manufacturer's instructions. Briefly, 500ng of total RNA was ribo depleted, fragmented and went under first and second strand synthesis, A tailing, adapter ligation and PCR amplification (using 11 cycles). Final libraries were quantified using the KAPA Library Quantification Kit (KAPA Biosystems), Qubit Fluorometer (Life Technologies) and Agilent 2100 BioAnalyzer, and were sequenced on an Illumina HiSeq2500 sequencer (v4 chemistry) using 1 x 100bp cycles generating 25-30 million reads.

<u>Flow Cytometry antibodies used in LPL staining</u>

Antibodies CD3e (clone 145-2C11, Biolegend), CD4 (clone L3T4, Biolegend), CD11b (clone M1/70, Biolegend), CD11c (clone N418, Biolegend), CD45 (clone 30F11, Biolegend), CD64 (clone X54-5/7.1, BD), CD103 (clone 2E7, eBioscience), F4/80 (clone CI: A3-1, Biolegend), IFN-γ (clone XMG1.2, eBioscience), IL-17A (clone eBio17B7, eBioscience). ALDEFLUOR staining kit (Stem cell technologies) was used according to the manufacturer instructions (Supplementary Table 36).

Supplementary References

139 Di Narzo, A. F. *et al.* Blood and Intestine eQTLs from an Anti-TNF-Resistant Crohn's Disease Cohort Inform IBD Genetic Association Loci. *Clinical and translational gastroenterology* **7**, e177, doi:10.1038/ctg.2016.34 (2016).

140 Kundaje, A. *et al.* Integrative analysis of 111 reference human epigenomes. *Nature* **518**, 317-330, doi:10.1038/nature14248 (2015).

141 Maurano, M. T. *et al.* Systematic localization of common disease-associated variation in regulatory DNA. *Science (New York, N.Y.)* **337**, 1190-1195, doi:10.1126/science.1222794 (2012).

142 Mokry, M. *et al.* Many inflammatory bowel disease risk loci include regions that regulate gene expression in immune cells and the intestinal epithelium. *Gastroenterology* **146**, 1040-1047, doi:10.1053/j.gastro.2013.12.003 (2014).

143 Roussos, P. *et al.* A role for noncoding variation in schizophrenia. *Cell reports* **9**, 1417-1429, doi:10.1016/j.celrep.2014.10.015 (2014).

144 Yang, X. *et al.* Validation of candidate causal genes for obesity that affect shared metabolic pathways and networks. *Nature genetics* **41**, 415-423, doi:10.1038/ng.325 (2009).

145 Uhlig, H. H. *et al.* The diagnostic approach to monogenic very early onset inflammatory bowel disease. *Gastroenterology* **147**, 990-1007.e1003, doi:10.1053/j.gastro.2014.07.023 (2014).

146 Kabakchiev, B. & Silverberg, M. S. Expression quantitative trait loci analysis identifies associations between genotype and gene expression in human intestine. *Gastroenterology* **144**, 1488-1496, 1496.e1481-1483, doi:10.1053/j.gastro.2013.03.001 (2013).

147 Greenawalt, D. M. *et al.* A survey of the genetics of stomach, liver, and adipose gene expression from a morbidly obese cohort. *Genome research* **21**, 1008-1016, doi:10.1101/gr.112821.110 (2011).

148 Ferraro, A. *et al.* Interindividual variation in human T regulatory cells. *Proceedings of the National Academy of Sciences of the United States of America* **111**, E1111-1120, doi:10.1073/pnas.1401343111 (2014).

149 Fairfax, B. P. *et al.* Genetics of gene expression in primary immune cells identifies cell type-specific master regulators and roles of HLA alleles. *Nature genetics* **44**, 502-510, doi:10.1038/ng.2205 (2012).

150 Fairfax, B. P. *et al.* Innate immune activity conditions the effect of regulatory variants upon monocyte gene expression. *Science (New York, N.Y.)* **343**, 1246949, doi:10.1126/science.1246949 (2014).

151 Lee, M. N. *et al.* Common genetic variants modulate pathogen-sensing responses in human dendritic cells. *Science (New York, N.Y.)* **343**, 1246980, doi:10.1126/science.1246980 (2014).

152 Raj, T. *et al.* Polarization of the effects of autoimmune and neurodegenerative risk alleles in leukocytes. *Science (New York, N.Y.)* **344**, 519-523, doi:10.1126/science.1249547 (2014).

153 Schramm, K. *et al.* Mapping the genetic architecture of gene regulation in whole blood. *PloS one* **9**, e93844, doi:10.1371/journal.pone.0093844 (2014).

154 Westra, H. J. *et al.* Systematic identification of trans eQTLs as putative drivers of known disease associations. *Nature genetics* **45**, 1238-1243, doi:10.1038/ng.2756 (2013).

155 Colantuoni, C. *et al.* Temporal dynamics and genetic control of transcription in the human prefrontal cortex. *Nature* **478**, 519-523, doi:10.1038/nature10524 (2011).

156 Gibbs, J. R. *et al.* Abundant quantitative trait loci exist for DNA methylation and gene expression in human brain. *PLoS genetics* **6**, e1000952, doi:10.1371/journal.pgen.1000952 (2010).

157 Grundberg, E. *et al.* Mapping cis- and trans-regulatory effects across multiple tissues in twins. *Nature genetics* **44**, 1084-1089, doi:10.1038/ng.2394 (2012).

158 Human genomics. The Genotype-Tissue Expression (GTEx) pilot analysis: multitissue gene regulation in humans. *Science (New York, N.Y.)* **348**, 648-660, doi:10.1126/science.1262110 (2015).

159 Rosenbloom, K. R. *et al.* ENCODE whole-genome data in the UCSC Genome Browser: update 2012. *Nucleic acids research* **40**, D912-917, doi:10.1093/nar/gkr1012 (2012).

160 Andersson, R. *et al.* An atlas of active enhancers across human cell types and tissues. *Nature* **507**, 455-461, doi:10.1038/nature12787 (2014).

161 Hnisz, D. *et al.* Super-enhancers in the control of cell identity and disease. *Cell* **155**, 934-947, doi:10.1016/j.cell.2013.09.053 (2013).

162 Schmidt, S. V. *et al.* The transcriptional regulator network of human inflammatory macrophages is defined by open chromatin. *Cell research* **26**, 151-170, doi:10.1038/cr.2016.1 (2016).

163 Abecasis, G. R. *et al.* An integrated map of genetic variation from 1,092 human genomes. *Nature* **491**, 56-65, doi:10.1038/nature11632 (2012).

164 Schork, A. J. *et al.* All SNPs are not created equal: genome-wide association studies reveal a consistent pattern of enrichment among functionally annotated SNPs. *PLoS genetics* **9**, e1003449, doi:10.1371/journal.pgen.1003449 (2013).

165 Lango Allen, H. *et al.* Hundreds of variants clustered in genomic loci and biological pathways affect human height. *Nature* **467**, 832-838, doi:10.1038/nature09410 (2010).

166 Xue, J. *et al.* Transcriptome-based network analysis reveals a spectrum model of human macrophage activation. *Immunity* **40**, 274-288, doi:10.1016/j.immuni.2014.01.006 (2014).

167 Jubb, A. W., Young, R. S., Hume, D. A. & Bickmore, W. A. Enhancer Turnover Is Associated with a Divergent Transcriptional Response to Glucocorticoid in Mouse and Human Macrophages. *Journal of immunology (Baltimore, Md. : 1950)* **196**, 813-822, doi:10.4049/jimmunol.1502009 (2016).

168 McKinney, E. F., Lee, J. C., Jayne, D. R., Lyons, P. A. & Smith, K. G. T-cell exhaustion, co-stimulation and clinical outcome in autoimmunity and infection. *Nature* **523**, 612-616, doi:10.1038/nature14468 (2015).

169 Franzen, O. *et al.* Cardiometabolic risk loci share downstream cis- and trans-gene regulation across tissues and diseases.

170 Carcamo-Orive, I. *et al.* Analysis of Transcriptional Variability in a Large Human iPSC Library Reveals Genetic and Non-genetic Determinants of Heterogeneity. LID - S1934-5909(16)30401-5 [pii] LID - 10.1016/j.stem.2016.11.005 [doi].

171 Tusher, V. G., Tibshirani, R. & Chu, G. Significance analysis of microarrays applied to the ionizing radiation response. *Proceedings of the National Academy of Sciences of the United States of America* **98**, 5116-5121, doi:10.1073/pnas.091062498 (2001).

172 Li, J. & Tibshirani, R. Finding consistent patterns: a nonparametric approach for identifying differential expression in RNA-Seq data. *Statistical methods in medical research* **22**, 519-536, doi:10.1177/0962280211428386 (2013).
173 Ritchie, M. E. *et al.* limma powers differential expression analyses for RNA-sequencing and microarray studies. *Nucleic acids research* **43**, e47, doi:10.1093/nar/gkv007 (2015).
174 Law, C. W., Chen, Y., Shi, W. & Smyth, G. K. voom: Precision weights unlock linear model analysis tools for RNA-seq read counts. *Genome biology* **15**, R29, doi:10.1186/gb-2014-15-2-r29 (2014).
175 Smyth, G. K. Linear models and empirical bayes methods for assessing differential expression in microarray experiments. *Statistical applications in genetics and molecular biology* **3**, Article3, doi:10.2202/1544-6115.1027 (2004).
176 Millstein, J. & Volfson, D. Computationally efficient permutation-based confidence interval estimation for tail-area FDR. *Frontiers in genetics* **4**, 179, doi:10.3389/fgene.2013.00179 (2013).
177 Gautier, L., Cope, L., Bolstad, B. M. & Irizarry, R. A. affy--analysis of Affymetrix GeneChip data at the probe level. *Bioinformatics (Oxford, England)* **20**, 307-315, doi:10.1093/bioinformatics/btg405 (2004).
178 McCall, M. N. *et al.* The Gene Expression Barcode 3.0: improved data processing and mining tools. *Nucleic acids research* **42**, D938-943, doi:10.1093/nar/gkt1204 (2014).
179 Stojmirovic, A., Bliskovsky, A. & Yu, Y. K. CytoSaddleSum: a functional enrichment analysis plugin for Cytoscape based on sum-of-weights scores. *Bioinformatics (Oxford, England)* **28**, 893-894, doi:10.1093/bioinformatics/bts041 (2012).
180 Zhang, B. & Horvath, S. A general framework for weighted gene co-expression network analysis. *Statistical applications in genetics and molecular biology* **4**, Article17, doi:10.2202/1544-6115.1128 (2005).
181 Ravasz, E., Somera, A. L., Mongru, D. A., Oltvai, Z. N. & Barabasi, A. L. Hierarchical organization of modularity in metabolic networks. *Science (New York, N.Y.)* **297**, 1551-1555, doi:10.1126/science.1073374 (2002).
182 Langfelder, P. & Horvath, S. Eigengene networks for studying the relationships between co-expression modules. *BMC systems biology* **1**, 54, doi:10.1186/1752-0509-1-54 (2007).
183 Schadt, E. E. *et al.* Mapping the genetic architecture of gene expression in human liver. *PLoS biology* **6**, e107, doi:10.1371/journal.pbio.0060107 (2008).
184 Bussemaker, H. J., Foat Bc Fau - Ward, L. D. & Ward, L. D. Predictive modeling of genome-wide mRNA expression: from modules to molecules.
185 An integrated encyclopedia of DNA elements in the human genome. doi:D - NLM: NIHMS381381
D - NLM: PMC3439153.
186 Lee, E. & Bussemaker, H. J. Identifying the genetic determinants of transcription factor activity. doi:D - NLM: PMC2964119 EDAT- 2010/09/25 06:00 MHDA- 2011/01/13 06:00 CRDT- 2010/09/25 06:00 PHST- 2010/04/20 [received] PHST- 2010/06/20 [accepted] AID - msb201064 [pii] AID - 10.1038/msb.2010.64 [doi] PST - ppublish.
187 Lee, E., de Ridder J Fau - Kool, J., Kool J Fau - Wessels, L. F. A., Wessels Lf Fau - Bussemaker, H. J. & Bussemaker, H. J. Identifying regulatory mechanisms underlying tumorigenesis using locus expression signature analysis. doi:D - NLM: PMC3992641 OTO - NOTNLM.
188 Castaneda, F. E. *et al.* Targeted deletion of metalloproteinase 9 attenuates experimental colitis in mice: central role of epithelial-derived MMP. *Gastroenterology* **129**, 1991-2008, doi:10.1053/j.gastro.2005.09.017 (2005).
